# Gehua Jiecheng Decoction Inhibits Diethylnitrosamine-Induced Hepatocellular Carcinoma in Mice by Improving Tumor Immunosuppression Microenvironment

**DOI:** 10.3389/fphar.2020.00809

**Published:** 2020-05-29

**Authors:** Changpei Cheng, Qiyang Shou, Jiali Lang, Lu Jin, Xia Liu, Dongxin Tang, Zhu Yang, Huiying Fu

**Affiliations:** ^1^Affiliated First Hospital, Guizhou University of Traditional Chinese Medicine, Guiyang, China; ^2^Affiliated Secondary Hospital, Zhejiang Chinese Medical University, Hangzhou, China; ^3^Graduate School, Tianjin University of Traditional Chinese Medicine, Tianjin, China

**Keywords:** Gehua Jiecheng Decoction, diethylnitrosamine-induced hepatocarcinogenesis, immune microenvironment, inflammation, blood vessel formation

## Abstract

Gehua Jiecheng Decoction (GHJCD), a famous traditional Chinese medicine, has been used in the prevention and treatment of precancerous lesion of liver cancer, but its active mechanism has not been reported. This study aimed to evaluate the therapeutic effect of GHJCD on diethylnitrosamine (DEN)-induced hepatocellular carcinoma (HCC) in mice and the mechanism of this effect. We found that GHJCD effectively inhibited the occurrence of liver cancer and reduced the tumor area. The ratio of regulatory cells (Tregs), tumor-associated macrophages (TAMs), and myeloid-derived suppressor cells (MDSCs) in HCC microenvironment was down-regulated, whereas that of CD8 T and effective CD8 T cells was up-regulated. In addition, the expression levels of inflammatory factors IL-6, IL-10, TNF-α, and CCL-2 in the liver were inhibited, whereas those of the angiogenesis related molecules CD31 and VEGF were decreased. Moreover, WNT1, β-catenin, NF-kB, p-MAPK, p-AKT, and p-SRC content in the liver decreased, whereas APC content increased. These results suggested that GHJCD exerted a good inhibitory effect on liver cancer induced by DEN and thus may have a multi-target effect; GHJCD not only antagonized the immunosuppressive effect of the microenvironment of liver cancer but also exerted strong anti-inflammatory and antiangiogenesis effects.

## Introduction

Hepatocellular carcinoma (HCC) is the fifth most common cancer in the world and the third leading cause of cancer-related death (Siegel et al., 2018). In most countries, the mortality rate is almost equal to the morbidity rate, which indicates the lack of effective treatment for liver cancer. The molecular pathogenesis of HCC is extremely complex and heterogeneous. The traditional cancer treatment is mainly focused on a single target or a single mechanism. Unexpectedly, it has not achieved the ideal therapeutic effect. Or spectral anti-cancer produces greater toxicity and promotes the development of tumors (Xu et al., 2016). Systemic therapy is the best choice for patients with advanced liver cancer. Combination therapy of multiple treatment schemes is still the way to focus on in the future. Traditional Chinese medicine (TCM) shows a wide range of biological effects, and more evidence shows that it may be related to the regulation of tumor microenvironment and killing tumors by strengthening the immune system (Xu et al., 2016). Because of the effectiveness and fewer side effects of TCM, it has been widely accepted as a supplement and alternative therapy for cancer in China (Xiang et al., 2019).

As a new and effective tumor therapy, immunotherapy is considered to be one of the most promising areas of oncology, which aims to help patients’ own immune system resist cancer (Hu et al., 2019). In most patients with solid tumors, vascular abnormalities help the tumor evade attack by the immune system (Mukaida and Nakamoto, 2018). These abnormalities are due to an increase in angiogenic factors, such as VGEF (Bouzin et al., 2007) and angiogenin 2 (ANG2) (Etoh et al., 2000). Drugs targeting these molecules can normalize the abnormal tumor vascular system and increase the infiltration of immune effector cells. In addition, tumor-related inflammation destroys the anti-tumor immune response by promoting angiogenesis (Bouzin et al., 2007) and metastasis (Bollrath and Greten, 2009). Persistent inflammatory cells and factors can also transform tumor-related inflammatory microenvironment into immunosuppressive microenvironment, promoting tumor progression (Deng-Bo and Cui, 2010). Therefore, inflammation, angiogenesis, and immunosuppressive microenvironment are three major obstacles to tumor therapy.

Gehua Jiecheng Decoction (GHJCD) is derived from the theory of spleen and stomach written by Li Dongyuan, one of the four masters of medicine in the Yuan Dynasty. It is composed of green skin, wood incense, orange peel, ginseng, *Polyporus umbellatus*, Poria cocos, Fried Shenqu, Alisma, Ginger, Atractylodes macrocephala, Nutmeg kernel, and Pueraria lobate, Amomum villosum, a total of 13 TCMs. In China, GHJCD is often used to treat liver cirrhosis and liver injury caused by drinking (Hu et al., 2020). Pueraria lobata, the main component of GHJCD, has long been used in the treatment of chronic alcoholic liver injury, has a protective effect on alcohol-induced apoptosis, and there are no side effects of it (Wu et al., 2019). Pueraria lobata extracted reduces hepatic fibrosis and hepatotoxicity (Peng et al., 2019), inhibits the activation of Kupffer cells and weakens the anti-inflammatory response of NF-kB pathway (Ahmad et al., 2020). Ginseng has a strong inhibitory effect on inflammatory mediators such as IL-6 (IL-6) and TNF-α and proinflammatory cytokines induced by macrophages (Yao et al., 2019). 6-shogaol (6-sho), the bioactive component of ginger, has anti-inflammatory and anticancer properties, weakens the proliferation of tumor cells and induces the death of liver cancer cells (Nazim and Park, 2019). *Polyporus umbellatus* polysaccharide is the main bioactive component of its, which has the effects of immune enhancement, anti-tumor, anti-inflammation, and liver protection (Guo et al., 2019). Poria cocos polysaccharide is the main active component of its. Through the combined regulation of NF-kB signal transduction, it shows immunomodulatory activity can significantly reduce the tumor volume and has the pharmacological effect of anti-cancer (Tian et al., 2019). The racemic-dihydroguaiacic acid extracted from nutmeg showed effective cytotoxicity and anti-tumor activity in allogenic tumor-bearing mice (Thuong et al., 2014). Its water extract can inhibit the release of proinflammatory cytokines such as IL-6 and tumor necrosis factor (Kim et al., 2013). The water extract of *Amomum villosum* could significantly increase the percentage of CD4 T cells (Chen et al., 2018). *Atractylodes macrocephala* shows good cytotoxicity and anti-tumor effect by blocking S phase tumor cells. Especially compared with cyclophosphamide, it can protect immune organs better (Feng et al., 2019). Our previous study found that GHJCD can inhibit subcutaneous transplantation and orthotopic liver transplantation of HCC cells in mice (Guo et al., 2019). However, the active mechanism of GHJCD regulates liver cancer is still not clear.

This study is to observe the therapeutic effect of GHJCD on liver cancer induced by diethylnitrosamine (DEN), and to explore the mechanism of its anti-tumor activity, so as to provide theoretical basis for clinical treatment.

## Materials and Methods

### Animals and Reagents

Eighty male C3H mice (6 weeks old, weighing 20–25 g) were purchased from Beijing Weitong Lihua Experimental Animal Technology Co., Ltd. (Beijing, china), and kept in a specific pathogen free mouse breeding room with controlled temperature of 25 ± 1°C. The mice were provided free access to food.

Anti-CD45 FITC, anti-F4/80 PE, anti-CD11b APC, and anti-Gr1PerCP-Cy5.5 were purchased from BD Biosciences (Lake Franklin, New Jersey, United States). Anti-CD3 FITC antibody was purchased from BioLegend (San Diego, California, United States). Primary antibodies against WNT1 (H-89) and NF-KB P65(F-6) were purchased from Santa Cruz Biotechnology Co., Ltd. (Santa Cruz Avenue, California, United States). Primary antibodies against VEGFA (VG-1), β-catenin, and APC were purchased from Abcam (Cambridge, MA, United States). Primary antibody against β-actin was purchased from Huaan Biotechnology Co., Ltd. (Hangzhou, China). Anti-CD31 was purchased from Abcam. DEN was purchased from Merck Group (Darmstadt, Germany).

### Liver Cancer Model

C3H mice were provided free access to water containing DEN 15 μg/ml, which was changed daily, for 23 weeks without interruption to successfully induce liver cancer in the mice.

### Experimental Groups

Eighty 6-week-old male C3H mice were randomly divided into four groups: the control, model, low-dose GHJCD (LG), and high-dose GHJCD (HG) groups. There were 20 mice in each group. For 23 weeks, mice in the control group were provided sterile water, whereas those in the other groups were provided DEN solution (15 μg/ml).

### Preparation of Extracts and Drug Treatment

GHJCD containing Flower of Pueraria montana var. lobata (Willd.) 15 g (Zhejiang Chinese Medical University prepared pieces Co., Ltd, Hangzhou, Zhejiang, China, No.: 190301), Nutmeg 15g (Hangzhou East China traditional Chinese Medicine prepared pieces Co., Ltd, Hangzhou, Zhejiang, China, No.: 190326), Fructus Amomi 15 g (Zhejiang Chinese Medical University prepared pieces Co., Ltd, Hangzhou, Zhejiang, China, No.: 190601), Alisma plantago-aquatica subsp. Orientale 6 g (Zhejiang Zoli Baicao traditional Chinese Medicine pieces Co., Ltd. Huzhou, Zhejiang, China, No.: 20190601), *Atractylodes Macrocephala* 6 g (Zhejiang Zoli Baicao traditional Chinese Medicine pieces Co., Ltd. Huzhou, Zhejiang, China, No.: 20190501), Panax ginseng C.A.Mey. (Araliaceae) 4.5 g (Zhejiang Chinese Medical University prepared pieces Co., Ltd, Hangzhou, Zhejiang, China, No.: 190601), *Polyporus umbellatus* 4.5 g (Hangzhou East China traditional Chinese Medicine prepared pieces Co., Ltd, Hangzhou, Zhejiang, China, No.: 190326), *Poria cocos* 4.5 g (Hangzhou East China traditional Chinese Medicine prepared pieces Co., Ltd, Hangzhou, Zhejiang, China, No.: 190830), Zingiber officinale Roscoe [Zingiberaceae] 6 g (Zhejiang Chinese Medical University prepared pieces Co., Ltd, Hangzhou, Zhejiang, China, No.: 190601), Pericarpium Citri Reticulatae 4.5 g (Hangzhou East China traditional Chinese Medicine prepared pieces Co., Ltd., Hangzhou, Zhejiang, China, No.: 190908), Pericarpium Citri Reticulatae Viride 0.9 g (Zhejiang Zoli Baicao traditional Chinese Medicine pieces Co., Ltd. Huzhou, Zhejiang, China, No.: 20190402), Radix Aucklandiae 1.5 g (Zhejiang Chinese Medical University prepared pieces Co., Ltd., Hangzhou, Zhejiang, China, No.: 181101), *Massa Medicata Fermentata* 6 g (Zhejiang Zoli Baicao traditional Chinese Medicine pieces Co., Ltd. Huzhou, Zhejiang, China, No.: 20190801). The above drugs were soaked in 500-ml aseptic water for 30 min, boiled for 30 min, and then filtered and concentrated into low-dose (crude drug concentration 2.25 g/ml) and high-dose (4.5 g/ml) GHJCD solutions.

Determination of components in water extract of GHJCD by UPLC-Q/TOF MS. The chromatographic conditions were as follows: the chromatographic column was Waters ACQUITY UPLC BEH C_18_ column (2.1 × 50 mm, 1.7 μ m, Waters, Milford, MA, USA); protection column was BEH C_18_ Van Guard (2.1 × 50 mm, 1.7 μ m, Waters, Milford, MA, USA); mobile phase: A solvent (methanol); B solvent (0.1% formic acid solution); flow rate: 0.25 ml; elution procedure: 0–0.5 min, 5%–45% A;10–15 min, 45%–70% A; 15–19 min,70%–100% A;, 5% A; 0.5–10 min. 19–20 min, 100% A; 20–21 min, 100–5% A; 21–24 min, 5% A. Column temperature: 40°C; sample temperature: 10°C; sample and standard sample injection volume: 0.5 μl.

Mass spectrometry conditions: SYNAPT G2-Si (Waters, Milford, MA, USA) mass spectrometer, electrospray ion source (ESI); positive and negative ion scanning mode, desolvation gas flow rate: 1,000 L, desolvation gas temperature: 500°C, ion source temperature: 120°C; Cone hole voltage: 20.0 V, capillary voltage: 3.0 kV (+), 3.0 kV (−); Locked mass solution: the on-line quality correction was carried out by Lockspray correction system of Waters company, leucine-enkephalin (Leueine-Enkephalin, [M + H]^+^ = 556.2771, [M − H] = 554.2615), the concentration of the solution was 2 ng/L and the flow rate was 10 μl. The scanning mode is MSe; mass scanning range: m50–1,200 Da, scanning time 0.2 s, positive and negative ion mode high collision energy transfer is 15–30 V, trap is 10 V, workstation: MassLynx V4.1 workstation. Data is matched and analyzed by Unifi 2.0 software (Waters, Milford, MA, USA).

The drugs were administered orally at a volume of 10 ml/kg once a day, and distilled water was administered at an equal volume. Three rats in each group were sacrificed at the 8th week, and six rats at the 12th week. The liver was removed, and liver sections were then stained with hematoxylin-eosin (HE) to observe the dynamic formation of liver cancer. After 23 weeks of treatment, the remaining mice were sacrificed. Before isolation of the liver, the hepatic portal vein was located by laparotomy and perfused with 10-ml normal saline to flush the liver until it turned gray.

### HE Staining of Liver Tissues

Mouse livers were cut into approximately 2.0 cm × 2.0 cm × 0.3 cm tissue blocks, fixed in 4% paraformaldehyde fixed solution for 24 h, dehydrated until transparent, embedded in wax, and then cut into 4-μm-thick sections. The sections were then subjected to Harris hematoxylin staining for 5 min, 1% hydrochloric acid ethanol color separation for 5 s, 0.5% ammonia for 20 s, and 0.5% eosin staining for 3 min. After each staining, the sections were washed in distilled water for 1 min. The sections were sealed after dehydration and transparent staining. The sections stained with HE were scanned, imaged, and observed by an Eclipse80i microscope (Nikon, Tokyo, Japan). The area of liver cancer was evaluated by NDP.view2 U12388Mui 01 digital pathological scanning software (Hamamatsu, Japan).

### Immunohistochemical Assay

Liver sections embedded in paraffin were dewaxed, subjected to antigen repair, incubated with 3% hydrogen peroxide for 10 min, blocked with 10% goat serum at 37°C for 10 min, incubated with CD31 antibody dilution with 1:500 at 37°C incubation for 2 h, and incubated with biotin-labeled secondary antibody at 37°C for 30 min. Next, the sections were incubated with horseradish enzyme-labeled streptavidin at 37°C for 30 min and mounted. The liver tissue sections were observed under a microscope with Ci-s positive image and text acquisition system (Nikon), analyzed by the ImageJ_v1.8.0 software (National Institutes of Health, Maryland).

### Flow Cytometry Analysis

The liver was placed into a 6-well plate and grinded. Next, lymphocytes were isolated using a lymphocyte separation solution, stained with the above antibodies, and detected by flow cytometry (Beckman Coulter, Pasadena, California).

### Cytometric Bead Array

A CBA Flex Set kit (BD Biosciences, San Jose, CA, United States) was used to assess interleukin-6 (IL-6), IL-10, interferon gamma (IFN-γ), TNF-α, and C-C motif chemokine ligand 2 (CCL2) levels in cell culture supernatant, according to the manufacturer’s instructions. Data were analyzed using the CellQuest software (BD Biosciences) and BD Pharmingen (BD Biosciences).

### Western Blotting Analysis

First, 100-mg liver tissue was cut, mixed with 350-µl lysate (Biyuntian Biotechnology Co., Ltd., Shanghai, China), ground on ice, and centrifuged (12,000 rpm at 4°C for 10 min). The supernatant was taken, and the total protein in liver tissue was extracted. Protein concentration in liver tissue was detected by BCA protein concentration determination kit (Biyuntian Biotechnology Co., Ltd.). The total protein was then separated by 8% or 10% SDS gel electrophoresis according to the molecular weight of the protein, and transferred to a nitrofibrin-imprinted membrane. The nitrofibrin imprinted membrane was blocked by 5% skim milk in Tris buffer for 2 h, and then incubated for 24 h with anti-NF-kB, WNT1, β-catenin, VEGF, and APC (1:1,000 dilution) at 4°C. The membrane was then washed three times with washing buffer for 10 min, incubated with secondary antibodies at room temperature for 2 h, washed three times for 10 min each, and treated with Millipore Western Blot HRP chemiluminescence solution (Millipore Corporation, Billerica, MA, USA). Protein visualization was carried out with an ultra-sensitive chemiluminescence imaging system (A Biotechne Brand, United States), and then the strip was quantitatively analyzed by the AlphaView-FluorChem FC3 3.4.0.0 software (ProteinSimple, Silicon Valley, California).

### Statistical Analysis

Differences between mean values of normally distributed data were evaluated by one-way analysis of variance (ANOVA) using the statistical package for the social sciences (SPSS) 18.0 software (SPSS Inc., Chicago, IL, USA). All the data were expressed as mean ± standard error of mean (SEM).

## Results

### Determination of GHJCD Water Extract by UPLC-Q/TOF MS

The water extract of GHJCD was detected by UPLC-Q/TOF MS method, and the results showed that there were 60 components in the water extract, many of which came from Flower of Pueraria montana var. lobata (Willd.), the “monarch medicine” of GHJCD, and the others from Panax ginseng C.A.Mey. (Araliaceae), *Atractylodes Macrocephala* and so on (**Table 1**).

**Table 1:** Characteristics of some chemical components in GHJCD by UPLC-Q/TOF MS analysis.

Name	Formula	Molecular weight	RT (min)	mzCloud BestMatch
New hesperidin	C28H34O15	610.1948	7.85	99.59
Puerarin	C21H20O9	416.1146	6.82	99.98
Genistein glycoside	C21H20O10	432.1094	8.82	99.96
Puerarin-4′-O-glucoside	C27H30O14	578.168	5.68	99.97
Narirutin	C27H32O14	580.1834	6.06	96.95
Puerarin celery glucoside	C26H28O13	548.1576	7.48	99.89
3′-methoxydaidzein	C16H12O5	284.0707	5.82	99.96
Daidzein	C15H10O4	254.0603	6.48	99.89
Quercetin-3-o-α-l-rhamnoside	C21H20O11	448.105	6.72	96.92
Isoliquiritigenin	C15H12O4	256.0762	7.55	95.69
Isoflavone aglycone genistein	C15H10O5	270.0551	9.39	97.28
16 oxo Alisol A	C30H48O6	504.3492	12.21	97.87
3′-methoxysoybean glycoside	C22H22O10	446.1246	5.29	98.79
Vanillic acid-β -D-glucopyranosylester	C14H18O9	330.0975	2.52	94.56
Alpha cyperone	C15H22O	218.1689	15.32	96.69
Anthocyanin of awn handle	C16H12O4	268.0761	9.02	98.47
7-hydroxy-3,5,6,3′, 4′-pentamethoxyflavone	C20H20O8	388.1192	5.55	97.76
Naringenin	C15H12O5	272.0708	7.06	94.38
5 α, 8 α-cyclodioxo-(24R)-24- methylcholesterol-6,9 (11), 22-triene-3 β-ol	C28H42O3	426.3171	14.21	97.57
Dihydroxylene lactone	C15H22O2	234.1634	12.94	94.27
Alismatin B	C15H26O5S	318.1505	16.87	98.17
Mycosterone B	C28H44O6	476.3182	11.02	98.46
Alismatin A	C15H26O4S	302.3582	13.94	95.73
24-acetyl Alisol E	C32H52O6	532.3719	19.45	96.42
Alismatin C	C15H24O4S	300.1399	15.33	97.24
Alisol C	C30H46O5	486.3355	12.96	96.42
Alismatine C	C15H24O2	236.1788	12.82	98.36
Alismatine A	C15H26O3	254.1885	11.08	98.25
Alismatine E	C15H26O3	254.189	10.94	97.13
Ginsenoside RF	C42H72O14	800.4925	10.3	98.47
Ginsenoside Rd	C48H82O18	946.5515	10.22	98.56
3,5,6,7,8,3′,4′ - heptanoxyflavone	C22H24O9	432.1423	12.53	97.35
5,6,7,4 ‘- tetramethoxyflavone	C19H18O6	342.1111	11.75	97.14
Kuzubutenolide A	C23H24O10	460.138	8.38	95.47
Anthocyanin of Stipa spinosa	C22H22O9	430.1266	7.57	97.52
7-hydroxy-3,5,6,8,3′,4′-hexamethoxyflavone	C21H22O9	418.1268	7.09	98.65
Chickpea sprout A	C16H12O5	284.0695	5.85	99.28
Daidzein-4′, 7-diglucoside	C27H30O14	578.1639	5.71	98.69
Ginsenoside Rg3	C42H72O13	784.4983	12.79	94.51
Ginsenoside Rb2	C53H90O22	1078.5956	14.84	94.13
Ginsenoside Rb1	C54H92O23	1108.6042	14.65	94.29
Ginsenoside Ro	C48H76O19	956.4999	14.26	96.31
Ginsenoside F1	C36H62O9	638.4389	12.9	95.25
3′-methoxypuerarin	C22H22O10	446.1213	5.32	92.68
Mycosterone D	C28H44O5	460.3142	19.44	94.18
Dibutyl phthalate	C16H22O4	278.1523	16.75	96.35
Gingerol	C17H26O4	294.184	11.79	98.36
Notoginsenoside R1	C47H80O18	932.5355	16.18	95.16
Poria cocos new acid DM	C32H48O6	528.3453	14.02	96.48
Emodin	C15H10O5	270.0536	6.87	89.39
Tangeritin	C20H20O7	372.1214	13.28	98.97
Sodium citrated	C21H22O8	402.132	12.16	88.56
Sinensetin	C20H20O7	372.121	11.1	88.24
Hesperidin	C28H34O15	610.1895	8.57	89.37
Hesperetin	C16H14O6	302.0797	7.87	88.47
Double Atractylodes macrocephala lactone	C30H38O4	462.276	15.26	87.45
3β-acetoxy atractylol	C17H22O3	274.1556	9.31	88.38
Hexadecanoic acid	C16H32O2	256.2407	14.46	87.36
Dehydrogladiolene	C15H22	202.1728	10.1	86.48
Japanese ginseng terpenoid ketone	C15H26O2	238.1922	3.93	87.34

### Inhibitory Effect of GHJCD on DEN-Induced HCC in Mice

**Figure 1:**
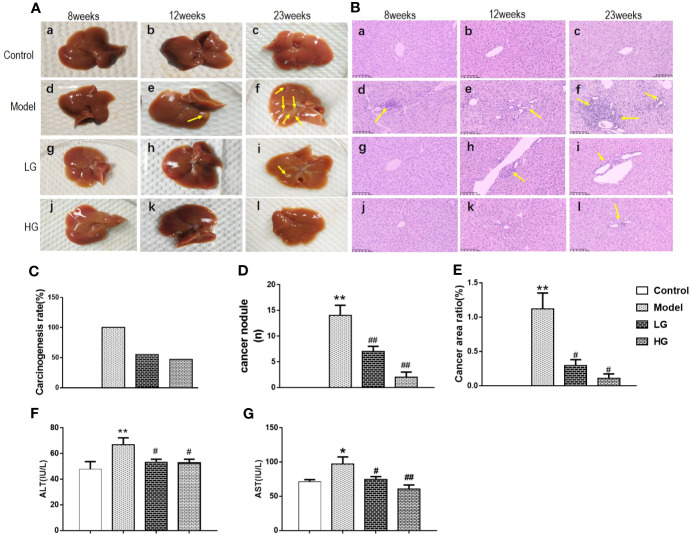
Inhibitory effect of GHJCD on diethylnitrosamine-induced hepatocellular carcinoma in mice. **(A)** General appearance of the liver was changed dynamically at the 8th, 12th, and 23rd week of experiment. **(B)** Pathological changes in HE-stained liver sections were observed at the 8th, 12th, and 23rd week of treatment. Scale bar: 250µm. **(C)** Carcinogenic rate of liver cancer: the ratio of the number of mice with at least one liver cancer lesion to that of experimental mice. **(D)** Number of liver cancer nodules: number of liver cancer lesions in the liver. **(E)** Cancer area ratio: the percentage of liver cancer lesion area and liver tissue section area. **(F)** Serum ALT level in mice. **(G)** The content of serum AST in mice. *p < 0.05, **p < 0.01 vs. control; ^#^p < 0.05, ^##^p < 0.01 vs. model.

After 8 weeks of treatment, no liver cancer nodules were observed in the liver of all mice (**Figure 1A**: a, d, g, j), and little sporadic punctate carcinoma was found in the model (**Figure 1B**: d).

After 12 weeks of treatment, there were obvious liver cancer nodules on the liver surface of the model mice (**Figure 1A**: e), but none were found in the control, LG, and HG groups (**Figure 1A**: b, h, k). HE staining assay showed that typical cancer nests were found in the model group mice (**Figure 1B**: e), and only localized punctate carcinogenesis occurred in the LG group (**Figure 1B**: h), while no cancerous changes in the HG and the control (**Figure 1B**: b, k).

As the experiment entered the final stage at the 23rd week, a large number of liver cancer nodules appeared on the liver surface of the model mice and merged into large liver cancer nodules (**Figure 1A**: f); moreover, a small amount of liver cancer nodules appeared on the liver surface of mice in the LG group (**Figure 1A**: i), but no liver cancer nodules were found on the liver surface of the control and HG groups (**Figure 1A**: c, l). HE staining analysis showed no cancer in the control mice (**Figure 1B**: c), but a large area of cancer nest was found in the model mice (**Figure 1B**: f). Although obvious cancer nests were also found in the LG group, the tumor area was much smaller than that in the model group (**Figure 1B**: i; **Figure 1D**), and only locally sporadic punctate carcinoma (**Figure 1B**: l) was found in the HG group.

The canceration rate was 100% in the model group, 55.56% in the LG group, and 47.37% in the HG group, and no cancer was found in the control group (**Figure 1C**). Hepatoma surface nodule data was performed with homogeneity test of variance, P = 0.001, P <0.05, the variance was not uniform, so after that, the Games-Howell test was used for multiple comparisons: the number of liver nodules in the model group was significantly higher than that in the control group (**Figure 1D**). Compared with those in the model group, the nodules in the LG and HG groups were significantly reduced, but the reduction was more significant in the HG group than in the LG group (**Figure 1D**).

Variance analysis is used to test the surface ratio data of liver cancer. First, we judge whether the variance is the same. Because P = 0.001, P < 0.05, the variance is not the same, so we use the Games-Howell test for multiple comparison: the cancerous area of the model was larger than that of the control, LG, and HG groups, with the HG group showing smaller cancerous area than the LG group (**Figure 1E**).

We detected the expression of Glutamic pyruvic transaminase (ALT) and Glutamic oxaloacetic transaminase (AST) in mouse serum. Compared with Control, ALT in Model was significantly higher, P < 0.01, while serum ALT in LG and HG was significantly lower than that in Model, P < 0.05 (**Figure 1F**).

Compared with control group, AST in model was significantly higher, with significant difference, P < 0.05; compared with model, AST in LG was significantly lower, with significant difference, P < 0.05; compared with model, AST in HG was significantly lower, with significant difference, P < 0.01 (**Figure 1G**).

### Immune Microenvironment of HCC Is Improved by GHJCD

**Figure 2:**
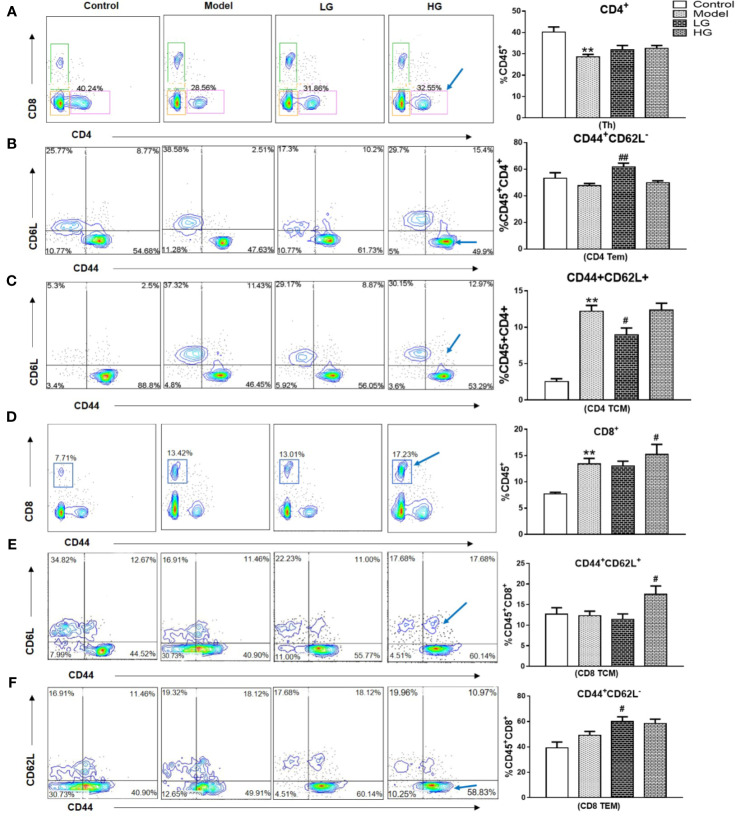
Expression of CD4^+^ and CD8^+^ in immune microenvironment of hepatocellular carcinoma at 23^th^ weeks. **(A)** CD4^+^ level in the liver. **(B)** CD4^+^Tem content in the liver. **(C)** CD4^+^ Tcm expression in the liver. **(D)** CD8^+^ level in the liver. **(E)** CD8^+^ Tcm level in the liver. **(F)** CD8^+^ Tem level in the liver. The CD4 ^+^, CD4^+^Tem, CD4^+^ Tcm, CD8^+^, CD8^+^ Tcm, CD8^+^ Tem data in the liver cancer microenvironment were analyzed by ANOVA. The homogeneity test was first performed, P > 0.05, the variances were uniform, and the LSD (L) test was used for multiple comparisons afterward, P < 0.05, the variances were not uniform. **p < 0.01 vs. control; ^#^p < 0.05, ^##^p < 0.01 vs. model.

The ratio of CD4^+^T cell (Th), CD4^+^Tem, CD4^+^Tcm, Cytotoxic T cells (CD8^+^T cells), CD8^+^Tcm, CD8^+^Tcm, CD8^+^ Regulatory T cell (Tregs), TAMs, Myeloid-derived suppressor cells (MDSCs) in the liver was detected by flow cytometry at the 23rd week of experiment. The expression of CD4^+^ decreased in the model; Low concentration of CHJCD increased the content of CD4Tem, but decreased CD4Tcm, and the expression of CD4Tcm in the model increased (**Figures 2A–C**).

The expression of CD8^+^ increased in the model (**Figure 2D**). Treatment with different concentrations of GHJCD led to different physiological activities: low-concentration GHJCD increased the level of CD8^+^ Tem (**Figure 2F**), whereas high-concentration GHJCD improved the function of CD8^+^ Tcm (**Figure 2E**).

**Figure 3:**
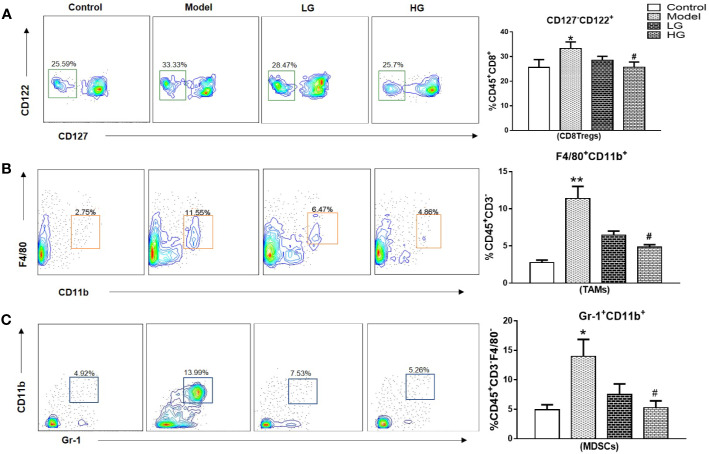
The content of CD8 ^+^ Tregs, TAMs, and MDSCs in the immune microenvironment of hepatocellular carcinoma at 23^th^ weeks. **(A)** CD8 ^+^ Tregs level in the liver. **(B)** TAMs content in the liver. **(C)** MDSCs expression in the liver. CD8 ^+^ Tregs, TAMs, and MDSCs data in the liver cancer microenvironment were analyzed by ANOVA. The homogeneity test was first performed, P > 0.05, the variances were uniform, and the LSD (L) test was used for multiple comparisons afterward, P < 0.05, the variances were not uniform. *p < 0.05, **p < 0.01 vs. control; ^#^p < 0.05, vs. model.

Immunosuppression of liver cancer microenvironment was very obvious. CD8^+^Tregs, TAMs, MDSCs increased significantly in the microenvironment of liver cancer, with obvious differences. However, the intervention of GHJCD led to the decrease of the expression of the above immunosuppressive factors, especially in the case of high concentration of GHJCD (**Figures 3A–C**).

### Inflammatory Factors and Chemokines in HCC Microenvironment Are Downregulated by GHJCD

**Figure 4:**
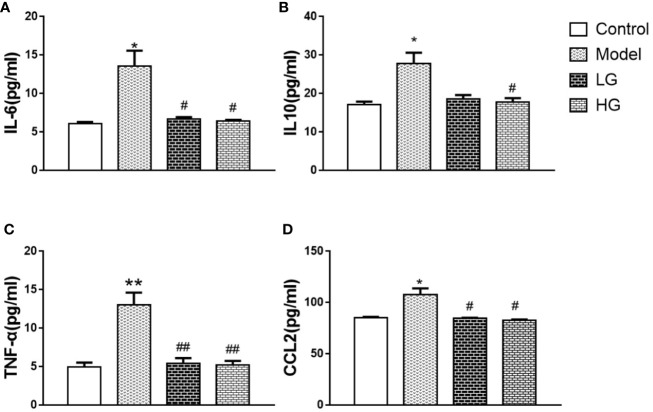
Inflammatory factors and chemokines in the microenvironment of hepatocellular carcinoma are down-regulated by GHJCD. **(A)** IL-6 content in liver tissue. **(B)** IL-10 content in liver tissue. **(C)** Expression of TNF-α in the liver. **(D)** CCl-2 content in liver tissue. IL-6, IL-10, TNF-α, and CCl-2 data in the liver cancer microenvironment were analyzed by ANOVA. The homogeneity test was first performed, P > 0.05, the variances were uniform, and the LSD (L) test was used for multiple comparisons afterward, P < 0.05, the variances were not uniform.*p < 0.05 vs. control; ^#^p < 0.05, ^##^p < 0.01 vs. model. Data were analyzed using single-variance *t*-test.

Compared with that in the control, IL-6 content was significantly increased in the model, but significantly decreased by GHJCD treatment in a dose-dependent manner (**Figure 4A**). IL-10 content also changed; it was significantly higher in the model group than in the control group, but significantly lower in the GHJCD-treated groups, especially in the HG group (**Figure 4B**). The TNF-α level in the LG and HG groups was significantly higher than that in the control group (**Figure 4C**), but significantly lower than that in the model (**Figure 4C**). The same trend was observed for CCL2 levels (**Figure 4D**).

### Angiogenic Ability of HCC in Its Microenvironment Is Inhibited by GHJCD

The enhancement of angiogenesis in tumor microenvironment is also the characteristic of occurrence and metastasis of liver cancer. We used WB and immunofluorescence techniques to detect the ability of angiogenesis in the microenvironment of HCC. We found that GHJCD significantly inhibited the ability of angiogenesis in HCC.

**Figure 5:**
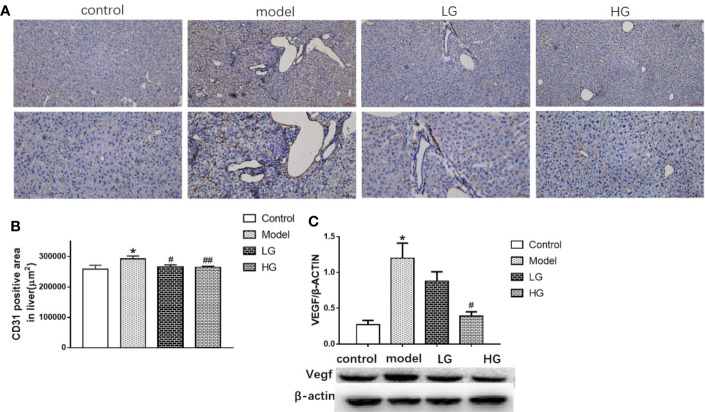
Angiogenic ability of hepatocellular carcinoma in microenvironment is inhibited by GHJCD. **(A)** Representative image of CD31 immunohistochemical staining in liver tissue. Top scale bar: 200 µm; bottom scale bar: 100 µm. **(B)** Area of CD31-positive distribution in liver tissue (µm^2^). **(C)** Expression of VEGF in liver tissue. CD31, VEGF data in the liver cancer microenvironment were analyzed by ANOVA. The homogeneity test was first performed, P > 0.05, the variances were uniform, and the LSD (L) test was used for multiple comparisons afterward, P < 0.05, the variances were not uniform.*p < 0.05, **p < 0.01 vs. control; ^#^p < 0.05 vs. model.

The expression of CD31 was detected by immunohistochemistry at the 23rd week of the experiment. The expression of CD31 in the model was significantly higher than that control (**Figures 5A, B**), LG was significantly reduced than that model (**Figure 5B**), especially in HG (**Figure 5B**).

Compared with the control group, the expression of VEGF protein in the model was significantly increased (**Figure 5C**), but compared with the model, the LG and HG groups gradually decreased, and the HG was more obvious (**Figure 5C**).

### GHJCD Downregulates NF-kB p65, WNT1, β-Catenin, Frz-7, P-MAPK, P-Akt, and P-src Expression, but Upregulates APC Expression in the Liver of DEN-Induced Liver Cancer Mice

**Figure 6:**
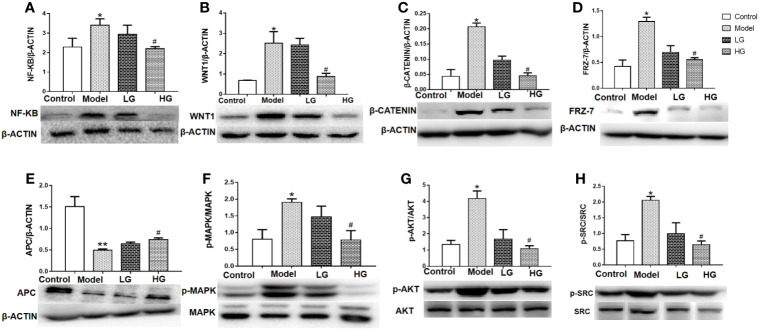
Expression of NF-kB p65, WNT1, β-catenin, Frz-7, APC, p-mapk/mapk, p-Akt/Akt, and p-src/src in the liver of mice with DEN-induced liver cancer. **(A)** Expression of NF-kB p65 in liver tissue. **(B)** Expression of WNT1 in liver tissue. **(C)** Expression of β-catenin in liver tissue. **(D)** Expression of Frz-7 in liver tissue. **(E)** Expression of APC in liver tissue. **(F)** Expression of p-MAPK/MAPK in liver tissue. **(G)** Expression of P-AKt/AKt in liver tissue.**(H)** Expression of p-src/src in liver tissue. NF-kB p65, WNT1, β-catenin, Frz-7, APC, p-mapk/mapk, p-Akt/Akt and p-src/src data in the liver cancer microenvironment were analyzed by ANOVA. The homogeneity test was first performed, P > 0.05, the variances were uniform, and the LSD (L) test was used for multiple comparisons afterward, P < 0.05, the variances were not uniform. *p < 0.05, **p < 0.01 vs. control; ^#^p < 0.05 vs. model. Data were analyzed using single-variance *t*-test.

NF-KB P65, WNT1, β-catenin, FRZ-7, APC, p-MAPK/MAPK, p-AKT/AKT, and p-SRC/SRC content in the liver was detected by western blotting. The protein levels of NF-kB, WNT1, β-catenin, p-MAPK/MAPK, p-AKT/AKT, and p-SRC/SRC in the liver of model mice were significantly higher than those in the control mice (P < 0.05, **Figures 6A–H**). With increasing drug concentration, NF-kB p65, WNT1, β-catenin, p-MAPK/MAPK, p-AKT/AKT, and p-SRC/SRC levels decreased in the liver of the LG and HG groups compared with those of the model group, but there was a significant difference in the HG group (**Figures 6A–H**). On the contrary, western blotting analysis showed that the level of APC protein in the model group was significantly lower than that in the control group (**Figure 6E**). GHJCD treatment, especially at a high concentration, significantly increased the expression of APC protein in the liver cancer microenvironment (**Figure 6E**).

## Discussion

In this study, we found that GHJCD could significantly improve the pathological features of liver cancer. The incidence of HCC in the model group was 100%, and the area ratio of HCC was significantly increased. In LG and HG groups treated with GHJCD, the incidence of cancer and nodular number of HCC were decreased dose-dependently, as well as the area ratio of HCC were reduced. GHJCD not only improved the microenvironment of HCC, including up-regulated the ratio of CD8+ T cells, down-regulated the ratio of Tregs, TAMs, and MDSCs, but also decreased the levels of inflammatory factors IL-6, IL-10, TNF-α, and CCl-2. In addition, GHJCD inhibited the angiogenic ability of liver cancer. These were also accompanied by down-regulation of single protein molecules in the inflammatory, such as NF-kB p65, and angiogenic signaling pathways, such as WNT1/β-catenin, Frz-7, APC, p-MAPK, p-AKT, and p-SRC.

The HCC model induced by DEN is a mature and widely used method to establish liver cancer in mice, and it is also a repeatable model of chronic liver injury, which is similar to that of human liver injury (Shou et al., 2015). For example, reactive oxygen species (ROS) production, compensatory proliferation, inflammation, and fibrosis (Guo et al., 2015). In our study, we found that liver cancer was successfully induced by DEN. In the model, there were no cancer nodules on the surface of the liver at the 8th week, but HE showed scattered canceration. Cancer nodules appeared on the surface of the liver at the 12th week of the experiment. A large number of cancer nodules were found on the surface of the liver at the end of the 23rd week of the experiment, combined with the cancer nest, and the canceration area was further expanded.

Immunosuppression is closely related to the occurrence and development of liver cancer. However, the liver’s unique immune tolerance microenvironment, liver cancer progression may have uncertain results. Persistent accumulation of cytotoxic T cells in liver cancer microenvironment and strong inhibition of liver tumors, and confers survival advantage to mice (Wen et al., 2019). By reshaping the tumor immune microenvironment, it can improve the anti-tumor immune response and inhibit the occurrence and development of liver cancer (Lei et al., 2018). It is well known that TAMS and MDSCs play a key role in the occurrence, deterioration, and metastasis of tumor in tumor microenvironment. TAMs promote the progress and metastasis of tumor cells by releasing a variety of cytokines, including chemokines, inflammatory factors, and growth factors (Jamieson et al., 2012). MDSC is a group of heterogeneous cells derived from bone marrow. It can significantly inhibit immune response and promote the formation of blood vessels such as (Lu et al., 2019). Treg cells suppress immune function in the immune system. Although the role of CD4^+^ CD25 ^+^regulatory Tregs in maintaining immune homeostasis has studied been extensively, recent findings indicate that CD8 ^+^ Tregs potentially play an immunomodulatory role in cancer (Dinesh et al., 2010). CD122^+^CD8^+^ Tregs are also an important regulatory T cell type and produce an anti-tumor immune response (Liu et al., 2006). T cells attack an infection in the body, and when the threat is over, the Treg cells signal the T cells to stop attacking. Cancer immunotherapy works by “overloading” the immune system to fight tumors. So when Tregs initiate signals that suppress the immune response, it inevitably blocks the effectiveness of immunotherapy. In our research, we found that the microenvironment of liver cancer was improved by GHJCD, the ratio of CD8 T cells in liver was up-regulated by GHJCD, while the ratio of TAMs and MDSCs, Tregs were down-regulated.

Inflammation is another important core factor in tumor microenvironment besides immunity. HCC is associated with chronic inflammation and fibrosis caused by different causes. The NF-κB pathway has an active role during inflammation (Wu et al., 2020) though factors such as p65, whose levels can reflect the extent of inflammation (Wang et al., 2020). P65 can aggravate inflammation by interacting with other signaling pathways such as the NF-κB/MAPK signaling pathway (Dong et al., 2020). The activation of NF-κ B may be a key step in the inflammatory cascade, which can induce the expression of IL-6 (Quay et al., 1998) TNF-α (Lee et al., 2016). Now, new findings suggest that chronic liver inflammation also promotes cancer by inhibiting immunity and increasing blood vessel formation (Ray, 2017). IL-10 is a pleiotropic cytokine, that appears to have contradictory roles (Mannino et al., 2015). For example, IL-10 not only down-regulates HLA-I but also up-regulates HLA-G, and causes immune escape in cancer cells (Urosevic and Dummer, 2003). IL-10 participates in the inflammatory response by inhibiting the bactericidal ability of polymorphonuclear leukocytes (PMN). If IL-10 is blocked, the ability to kill pathogenic microorganisms is enhanced (Siwapornchai et al., 2020). Furthermore, IL-10 is considered an immunosuppressive cytokine, which promotes tumor immune escape by reducing the anti-tumor immune responses in the tumor microenvironment. Additionally, the expression level of IL-10 is positively correlated with cancer recurrence (Li et al., 2019). In contrast, the accumulation of Tregs in cancer tissues requires high IL-10 expression (Kindlund et al., 2017). To understand the mechanisms by which IL-10 works, we propose to study drug-induced secretion of IL-10 in different types of cells in the future. Many inflammatory factors regulate immunosuppressive cells and affect the formation of blood vessels. Interleukin-6 (IL-6), IL-10 (Heim et al., 2015), and TNF (Xue et al., 2012) are important triggers of myeloid origin inhibiting the proliferation and recruitment of MDSCs and are considered to be the main coordinator of immunosuppressive tumor microenvironment (Zhang et al., 2018). Myeloid inhibitory cells promote tumor angiogenesis by increasing the expression of VEGF (Lu et al., 2019). In turn, VEGF can also promote the activity of MDSCs in HCC (Xu et al., 2016). So these evidences indicate that inflammation, immunosuppression, and angiogenesis affect and promote each other. Our results showed GHJCD not only inhibited the release of inflammatory cytokines, such as TNF-α, IL-10, and IL-6, but also inhibits the formation of blood vessels, the CD31 and VEGF protein expression. CD31 is also known as the platelet endothelial cell adhesion molecule. In immunohistochemistry, CD31 is mainly used to identify endothelial cells and evaluate tumor angiogenesis by correlating its expression levels to the extent of tumor growth (Sathornsumetee et al., 2008; Harmsen et al., 2019; Wang et al., 2019).

The WNT/β-catenin pathway is highly conserved and plays an important role in tumorigenesis, and it has been found to be overactivated in a variety of cancers. VEGF is one of the downstream of WNT/β-catenin pathway (Han et al., 2016). Moreover, the β-catenin/VEGF axis can promote tumor angiogenesis (Tang et al., 2019). APC protein is a negative regulator of WNT signaling pathway. It is encoded by tumor suppressor gene APC and binds to β-catenin, which prevents β-catenin from accumulating in cells and entering the nucleus, and promotes the degradation of β-catenin. The activation of WNT signal also can enhance the activity of antigen presenting cells and provide stimulation for Tregs (Mitkin et al., 2018). WNT/β-catenin pathway can regulate the function and differentiation of CD4, CD8 T cells, while blocking WNT/β-catenin pathway can inhibit Tregs (Dai et al., 2016). MAPK transmits signals from the cell surface to the nucleus and regulates a variety of physiological processes such as cell growth, differentiation, apoptosis and death (Yin et al., 2018). By regulating MAPK signaling pathway, TAMs can change from immunosuppressive M2-like phenotype to anti-tumor M1-like phenotype, increase the proportion of M1 macrophages, promote tumor recruitment of cytotoxic T lymphocytes (CTL) and inhibit angiogenesis (Deng et al., 2019). Akt is also associated with tumor increment and angiogenesis. Overexpression of VEGF mediated by Akt signal transduction can stimulate angiogenesis, which can be reduced by the use of inhibitor (Wang et al., 2019). SRC protein is also closely related to tumor angiogenesis. The transformation of SRC from inactivity to active conformation triggers the secretion of VEG through SRC/VEGF signal transduction pathway, which leads to the increase of tumor angiogenesis (Sun et al., 2018). In our study, GHJCD can significantly improve these protein molecules expressions, which suggests that GHJCD may inhibit angiogenesis and inflammation by these signaling pathways.

Exposure to high-risk factors such as DEN, which induces like precancerous lesions, is more likely to cause malignant tumors. In this experiment, GHJCD was administered to mice in the treatment group before the development of malignant tumors. The effective results confirmed that GHJCD can reduce the inevitable occurrence of malignant tumors, even after the occurrence of liver cancer, it can also effectively inhibit the development of tumor. In this study, GHJCD was found to significantly improve tumor microenvironment, including inhibiting inflammation and angiogenesis, and improving the infiltration of immunosuppressive cells. We know that these three factors themselves affect each other. Therefore, it is not clear whether GHJC has a direct effect on the three aspects at the same time, or whether it influences the other two factors through one factor. Next, we will explore the mechanism of inflammation, angiogenesis and immunosuppressive cells one by one.

**Figure 7:**
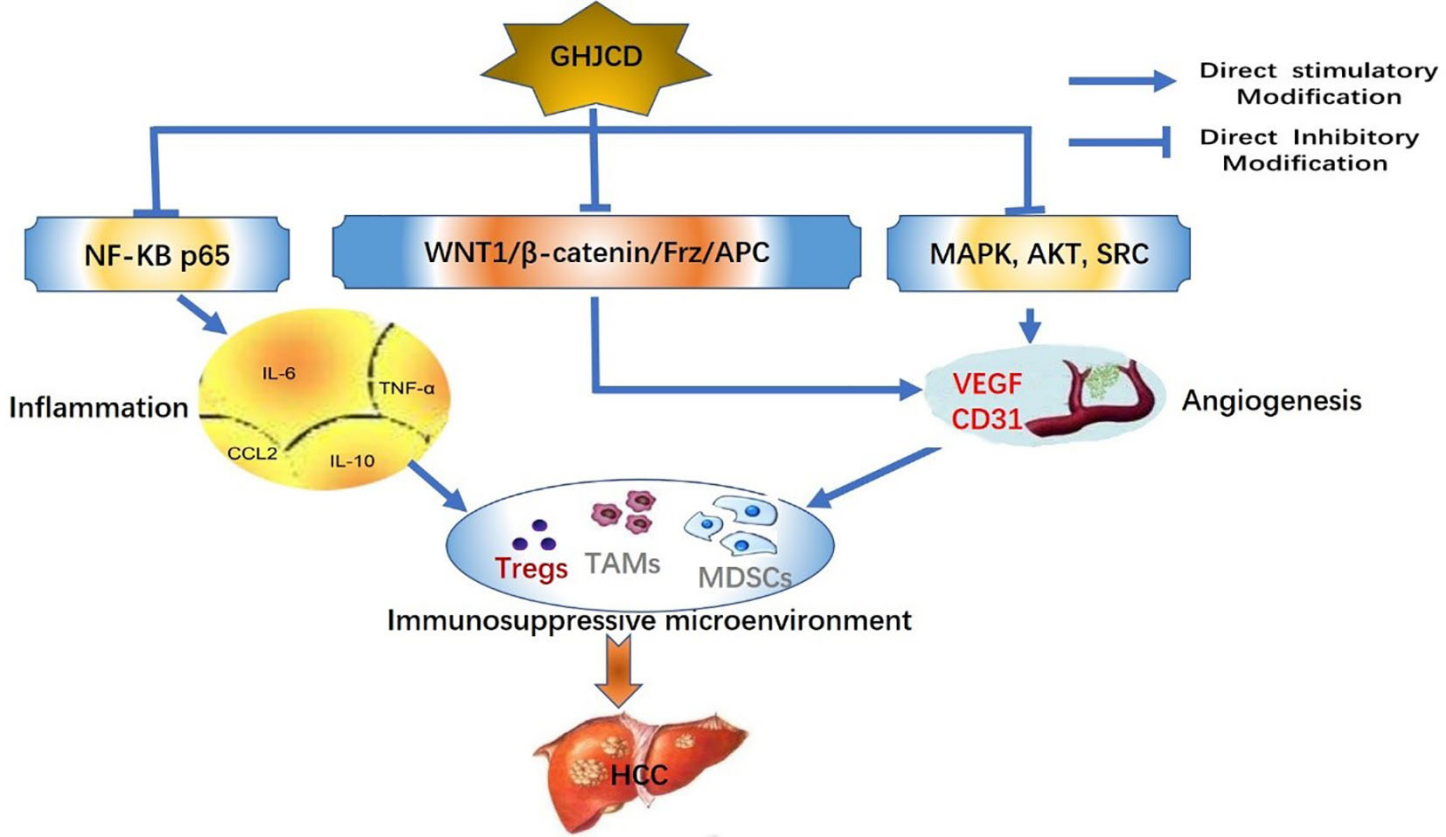
GHJCD inhibits HCC pathway by improving tumor microenvironment.

In conclusion, GHJCD has a significant inhibitory effect on DEN-induced liver cancer, and its anti-HCC pathway is extensive and multi-target. GHJCD not only has a strong anti-inflammatory effect, but also can inhibit the formation of blood vessels in liver cancer, resist the immunosuppressive effect in tumor microenvironment, and play the role of anti-liver cancer (**Figure 7**).

## Data Availability Statement

All datasets generated for this study are included in the article/**Supplementary Material**.

## Ethics Statement

The study was carried out in accordance with the recommendations of “Protection and use of Experimental Animals in the Animal Experimental Center of Zhejiang University of Traditional Chinese Medicine”. The program has been approved by the Animal Committee of the Animal Experimental Center of Zhejiang University of Traditional Chinese Medicine.

## Author Contributions

HF, QS, and ZY conceived and designed the study. QS, CC, LJ, and JL performed the experiments. HF and CC analyzed the data. HF, QS, and DT contributed reagents and materials. HF, XL and CC wrote the manuscript.

## Funding

This study was funded by the National Natural Science Foundation of China (Project Nos. 81673862, 81673645, 81873047, and 81573677), the Graduate Workstation Program of the Department of Education of Guizhou Province [Project Nos. JYSZ Word (2014) 018], Guizhou Provincial Department of Science and Technology High-level innovative Talent training Program Fund [100 levels, Project Nos. Qian Kehe Talents (2016) No.4032], Guizhou Provincial Organization Department of traditional Chinese Medicine tumor inheritance and Scientific and technological Innovation Talent Base Fund [Project Nos. Qian People Leading send (2018) 3], and Natural Science Foundation of Zhejiang Province (grant no. LQ17H030006).

## Conflict of Interest

The authors declare that the research was conducted in the absence of any commercial or financial relationships that could be construed as a potential conflict of interest.
