# Supplementary figures and images for: Gehua Jiecheng Decoction Inhibits Diethylnitrosamine-Induced Hepatocellular Carcinoma in Mice by Improving Tumor Immunosuppression Microenvironment
Source: Front Pharmacol. 2020 May 29;11:809. doi: 10.3389/fphar.2020.00809 (PMC7272686; doi:10.3389/fphar.2020.00809)

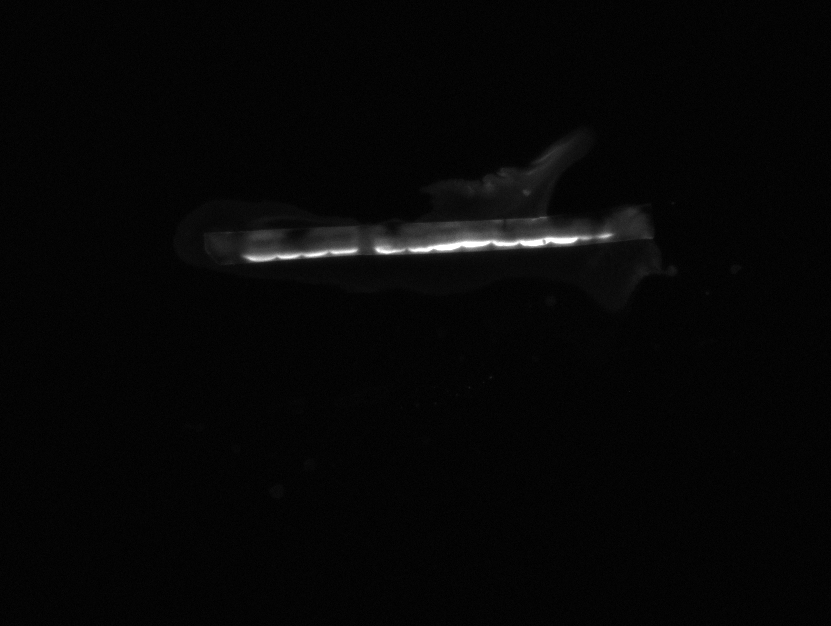

Supplement: Supplementary file 1 [file DataSheet_1.zip › WB1/2019-04-05_Apc cp/2019-03-13_b-act/2019-03-13_14-07-29_1_16bit.png]

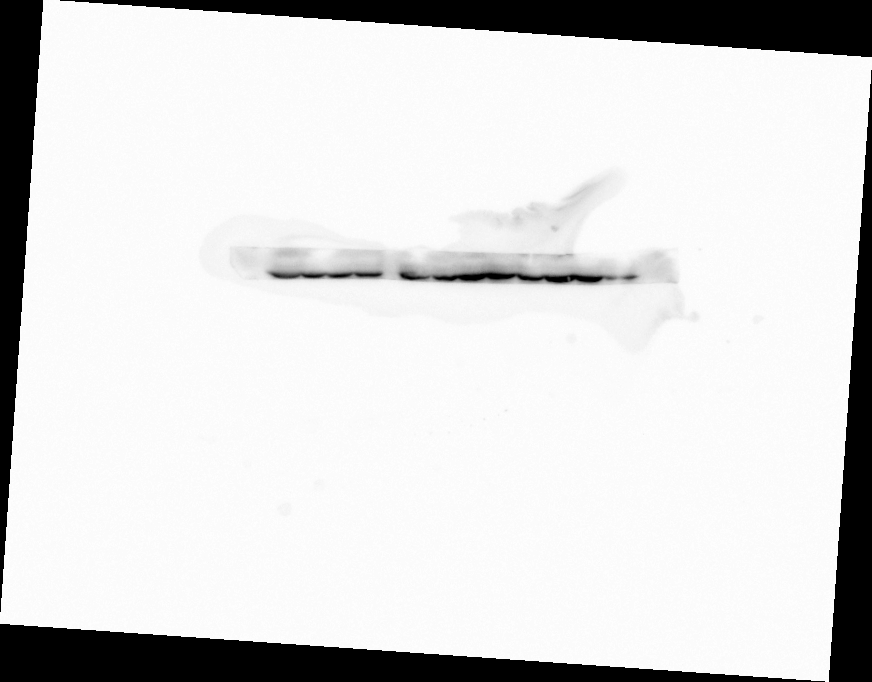

Supplement: Supplementary file 1 [file DataSheet_1.zip › WB1/2019-04-05_Apc cp/2019-03-13_b-act/2019-03-13_14-07-29_8bit - 8 bits 1.tif]

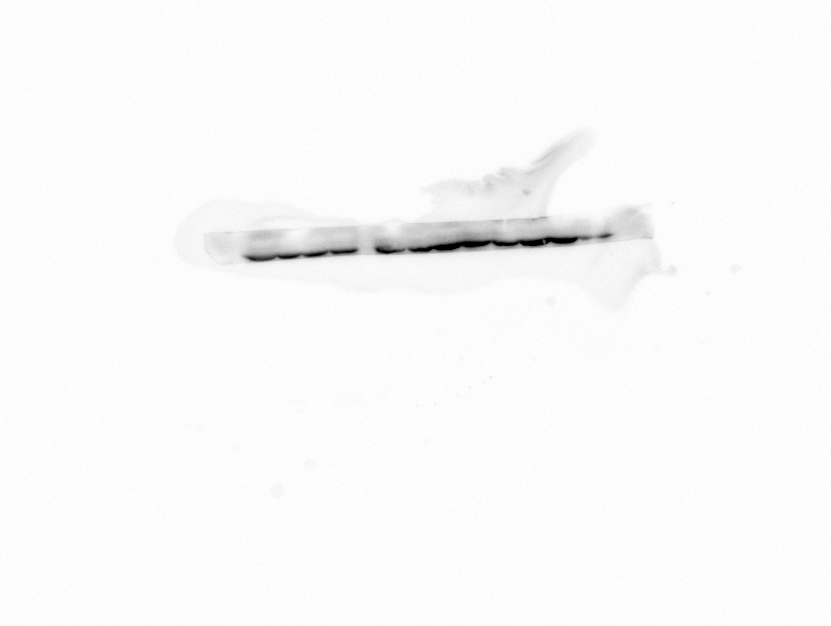

Supplement: Supplementary file 1 [file DataSheet_1.zip › WB1/2019-04-05_Apc cp/2019-03-13_b-act/2019-03-13_14-07-29_8bit.png]

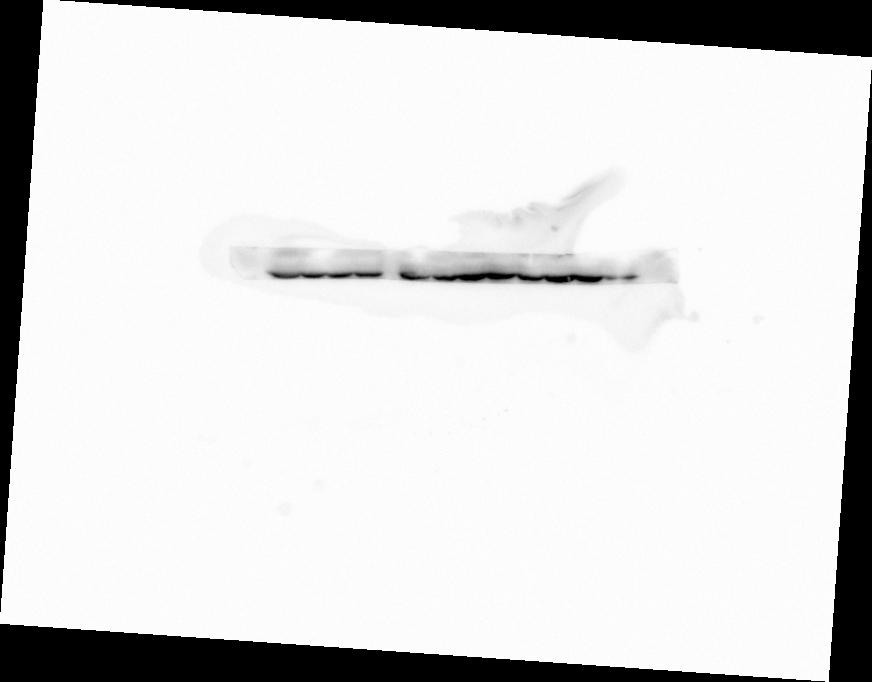

Supplement: Supplementary file 1 [file DataSheet_1.zip › WB1/2019-04-05_Apc cp/2019-03-13_b-act/2019-03-13_14-07-29_8bit.tif]

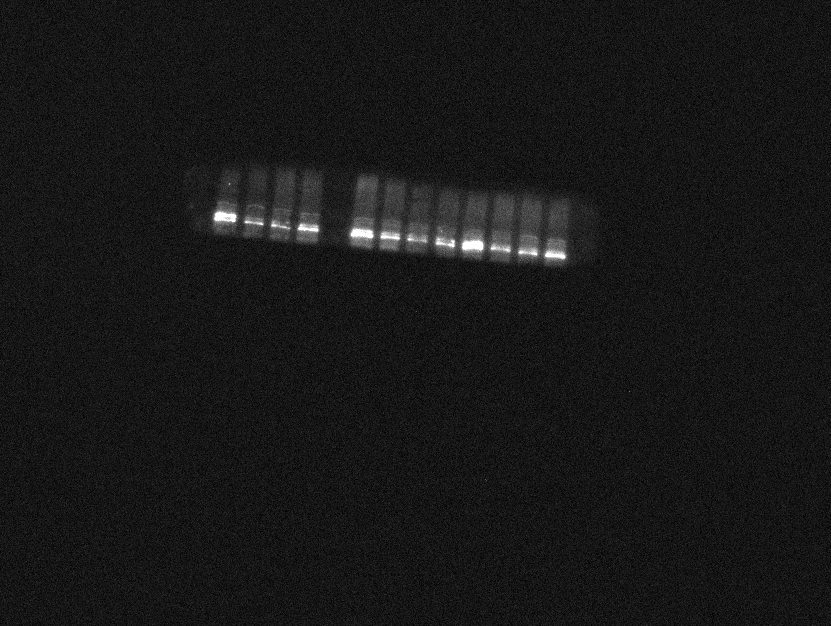

Supplement: Supplementary file 1 [file DataSheet_1.zip › WB1/2019-04-05_Apc cp/2019-04-05_Apc4 cp_1_16bit.png]

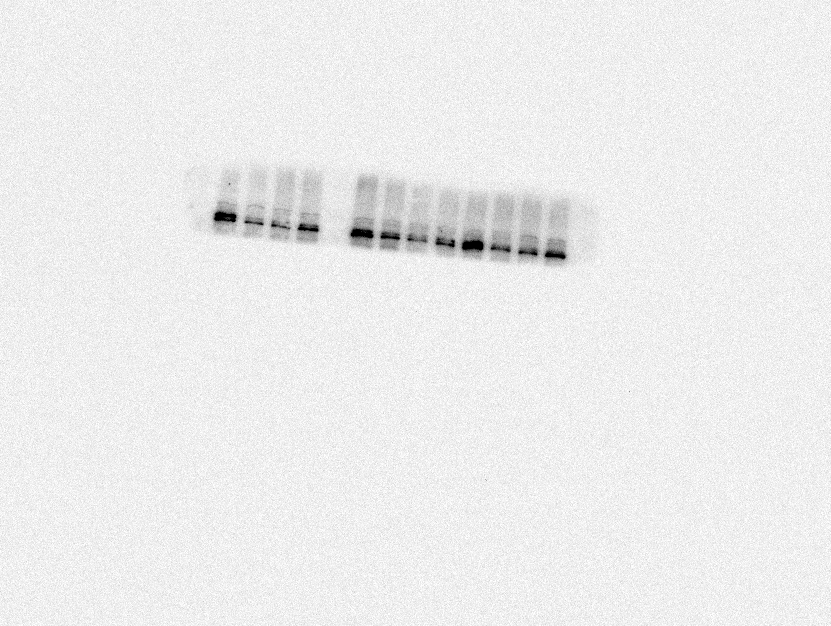

Supplement: Supplementary file 1 [file DataSheet_1.zip › WB1/2019-04-05_Apc cp/2019-04-05_Apc4 cp_8bit.png]

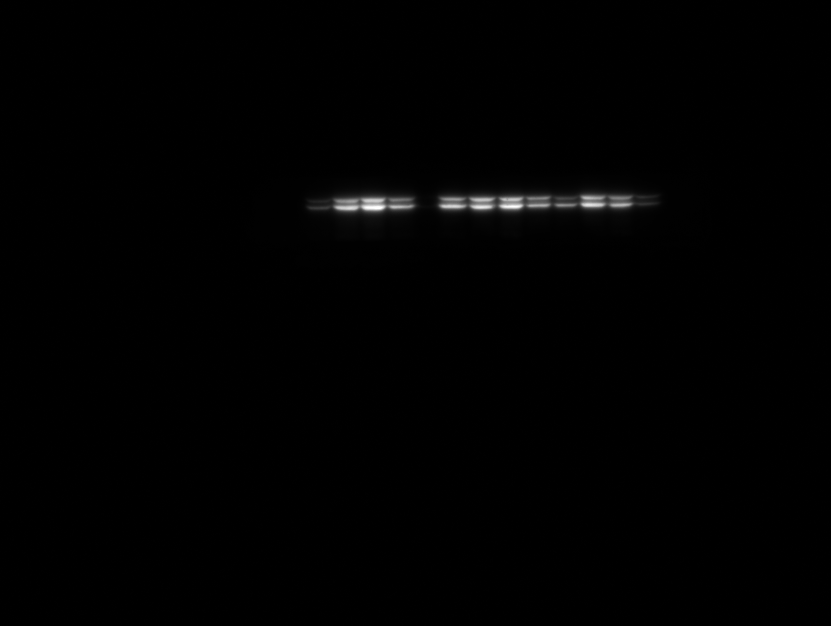

Supplement: Supplementary file 1 [file DataSheet_1.zip › WB1/2019-04-05_PmaPk/2019-04-05_PmaPk_1_16bit.png]

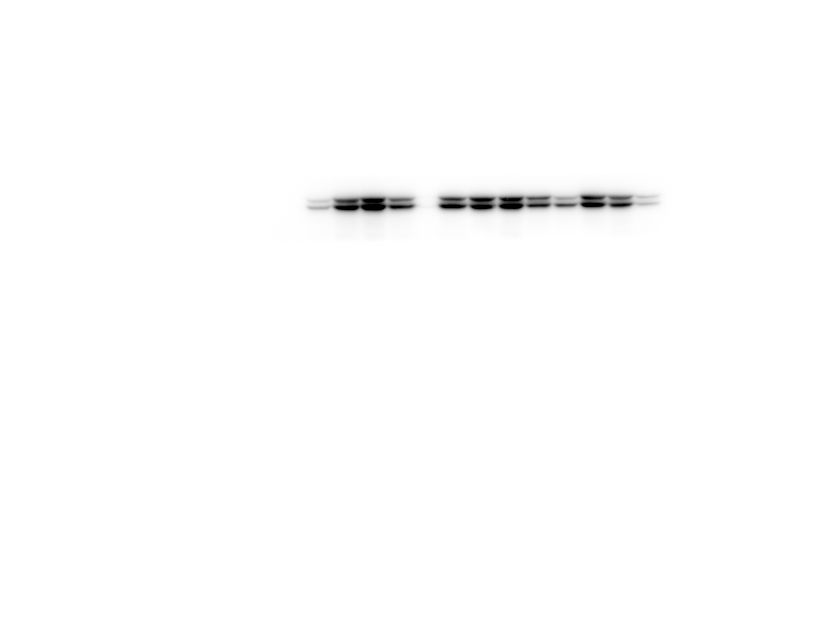

Supplement: Supplementary file 1 [file DataSheet_1.zip › WB1/2019-04-05_PmaPk/2019-04-05_PmaPk_8bit.png]

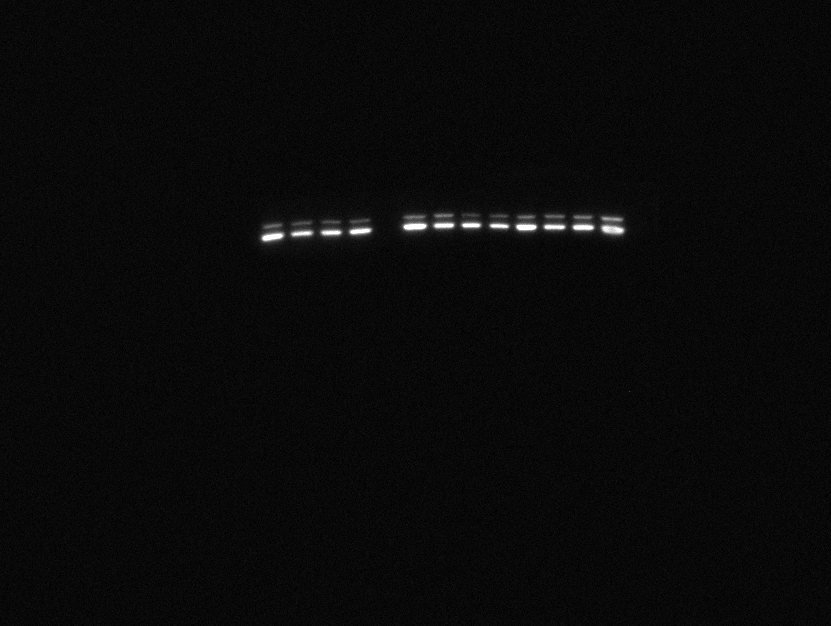

Supplement: Supplementary file 1 [file DataSheet_1.zip › WB1/2019-04-05_PmaPk/2019-04-08_Mapk cp/2019-04-08_Mapk cp_1_16bit.png]

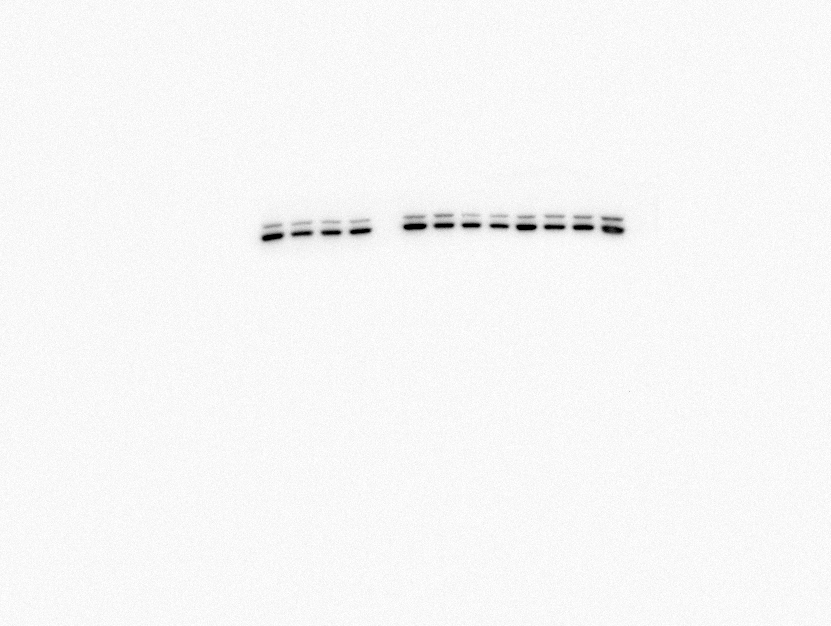

Supplement: Supplementary file 1 [file DataSheet_1.zip › WB1/2019-04-05_PmaPk/2019-04-08_Mapk cp/2019-04-08_Mapk cp_8bit.png]

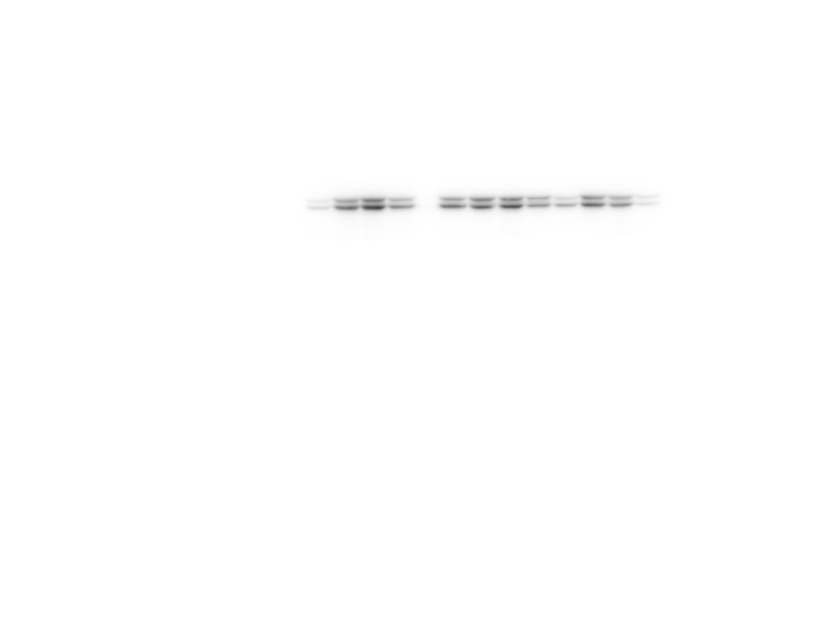

Supplement: Supplementary file 1 [file DataSheet_1.zip › WB1/2019-04-05_PmaPk/contrast/contrast_0.png]

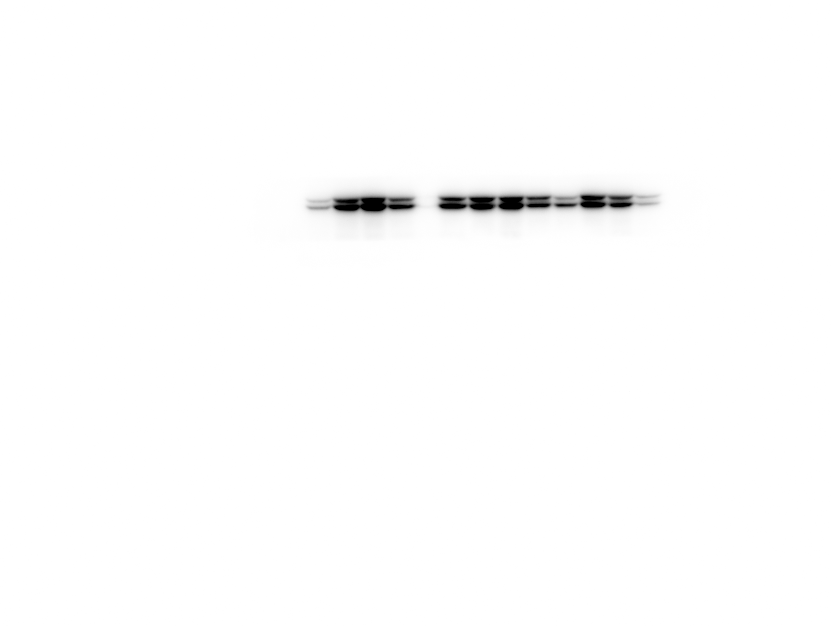

Supplement: Supplementary file 1 [file DataSheet_1.zip › WB1/2019-04-05_PmaPk/contrast/contrast_2.png]

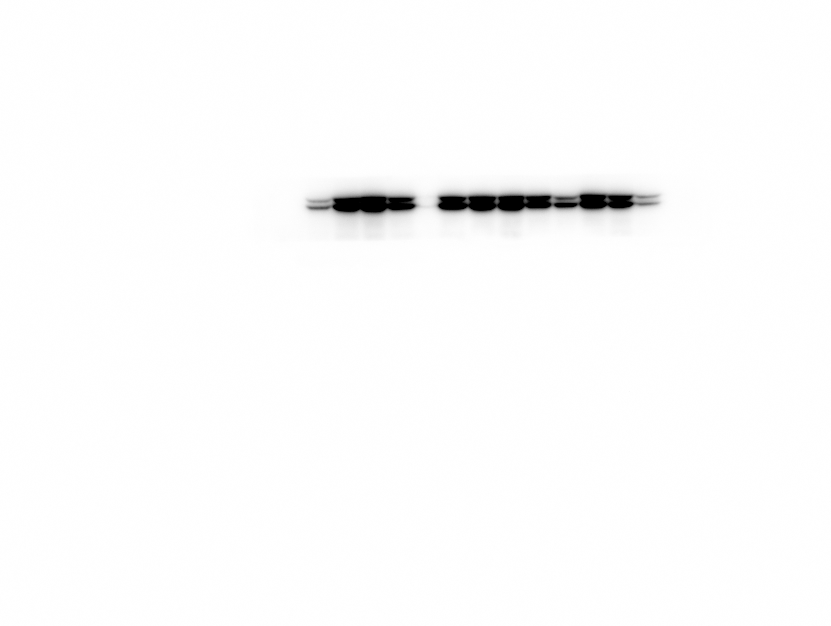

Supplement: Supplementary file 1 [file DataSheet_1.zip › WB1/2019-04-05_PmaPk/contrast/contrast_3.png]

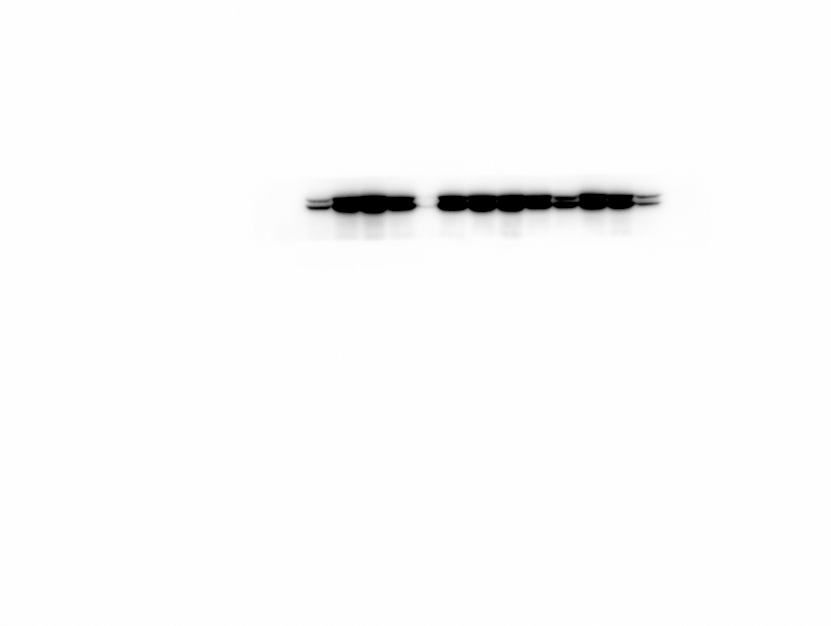

Supplement: Supplementary file 1 [file DataSheet_1.zip › WB1/2019-04-05_PmaPk/contrast/contrast_4.png]

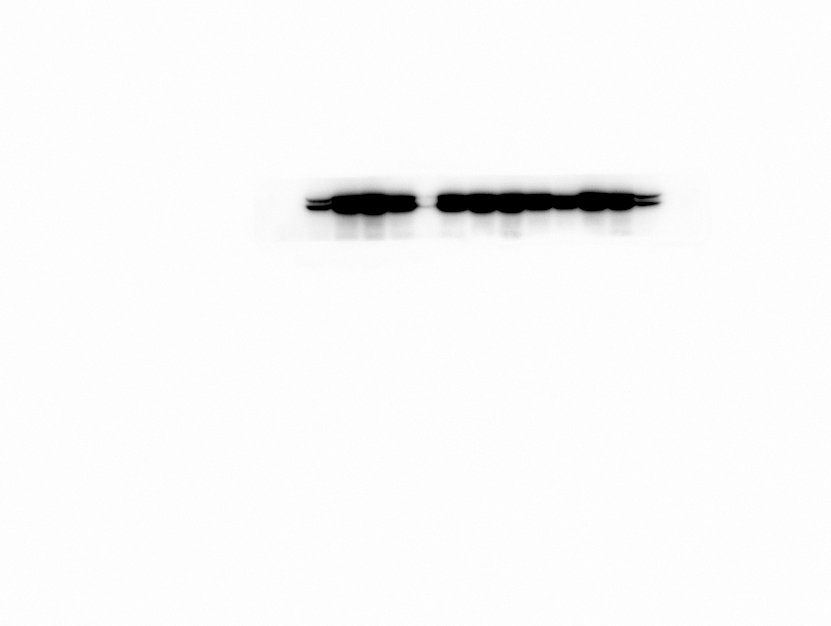

Supplement: Supplementary file 1 [file DataSheet_1.zip › WB1/2019-04-05_PmaPk/contrast/contrast_5.png]

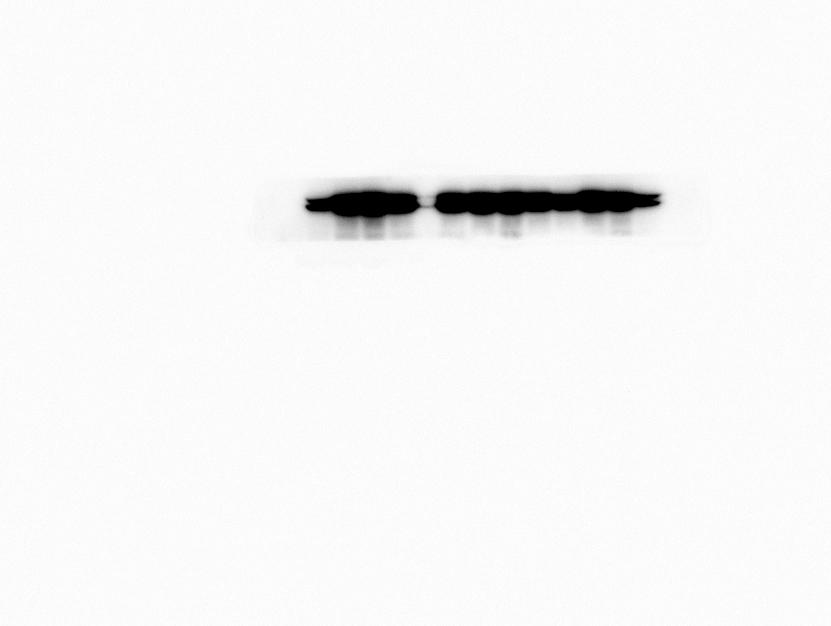

Supplement: Supplementary file 1 [file DataSheet_1.zip › WB1/2019-04-05_PmaPk/contrast/contrast_6.png]

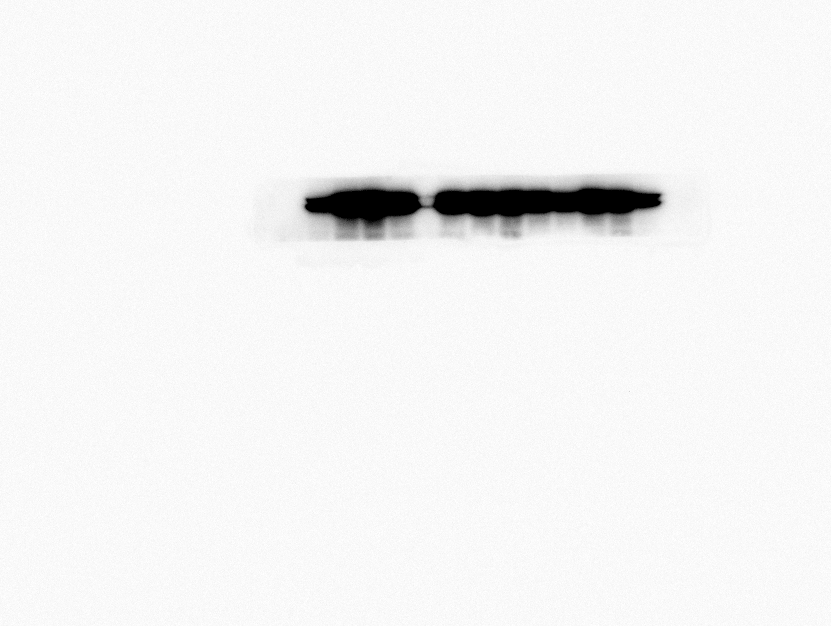

Supplement: Supplementary file 1 [file DataSheet_1.zip › WB1/2019-04-05_PmaPk/contrast/contrast_7.png]

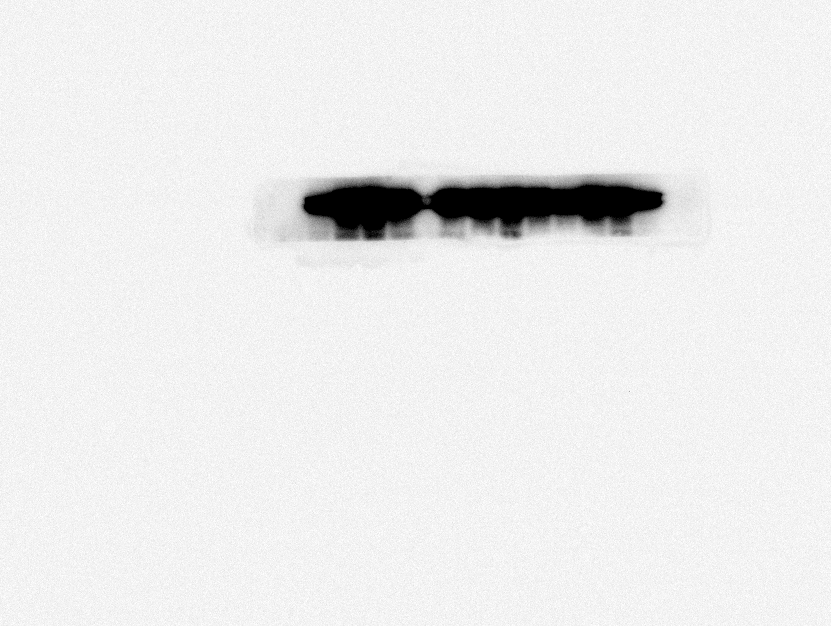

Supplement: Supplementary file 1 [file DataSheet_1.zip › WB1/2019-04-05_PmaPk/contrast/contrast_8.png]

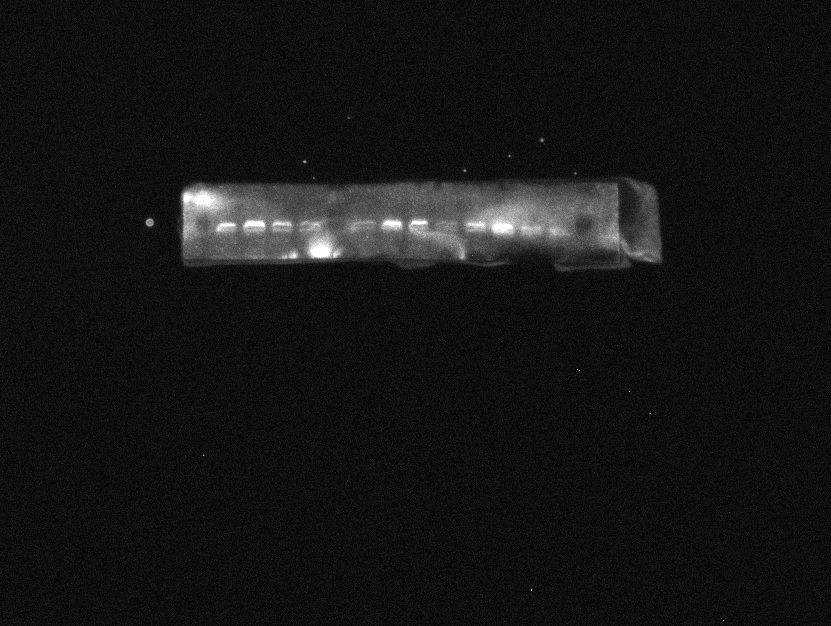

Supplement: Supplementary file 1 [file DataSheet_1.zip › WB1/2019-04-06_Nfkb/2019-04-06_16bit.png]

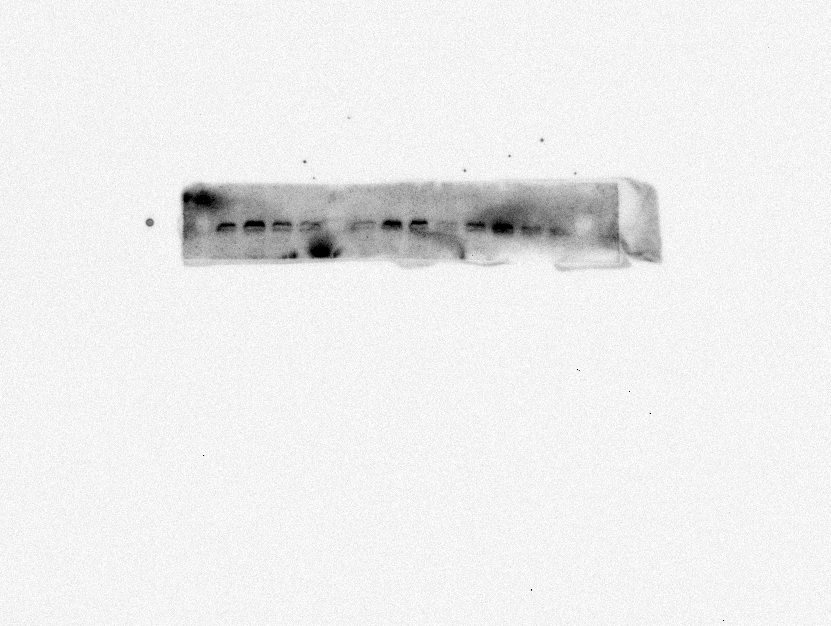

Supplement: Supplementary file 1 [file DataSheet_1.zip › WB1/2019-04-06_Nfkb/2019-04-06_Nfkb_8bit.png]

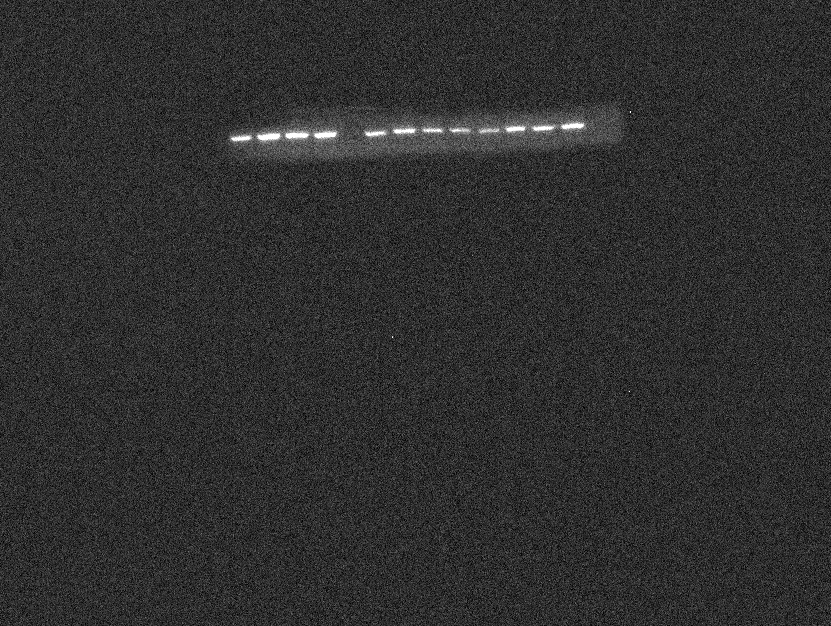

Supplement: Supplementary file 1 [file DataSheet_1.zip › WB1/2019-04-06_Nfkb/2019-04-06_b act cp/2019-04-06_b act cp_1_16bit.png]

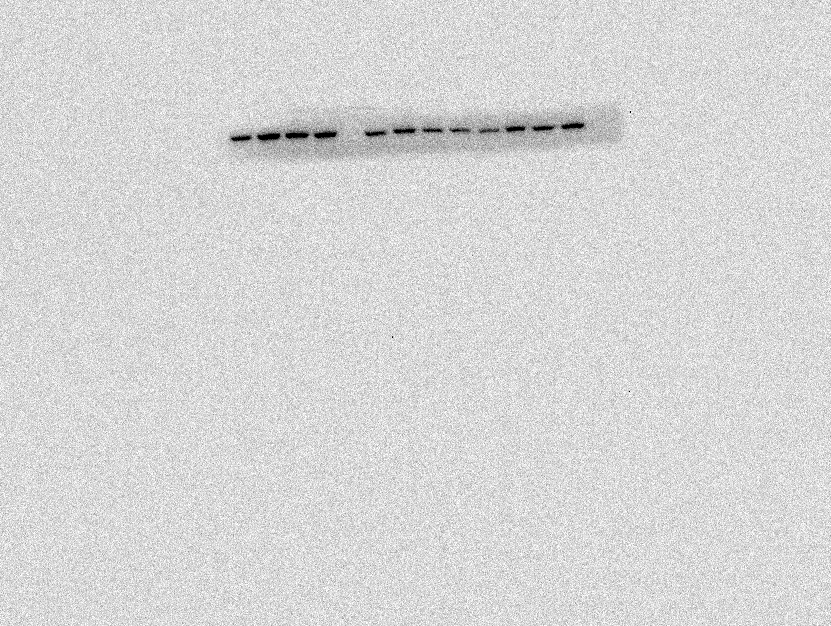

Supplement: Supplementary file 1 [file DataSheet_1.zip › WB1/2019-04-06_Nfkb/2019-04-06_b act cp/2019-04-06_b act cp_8bit.png]

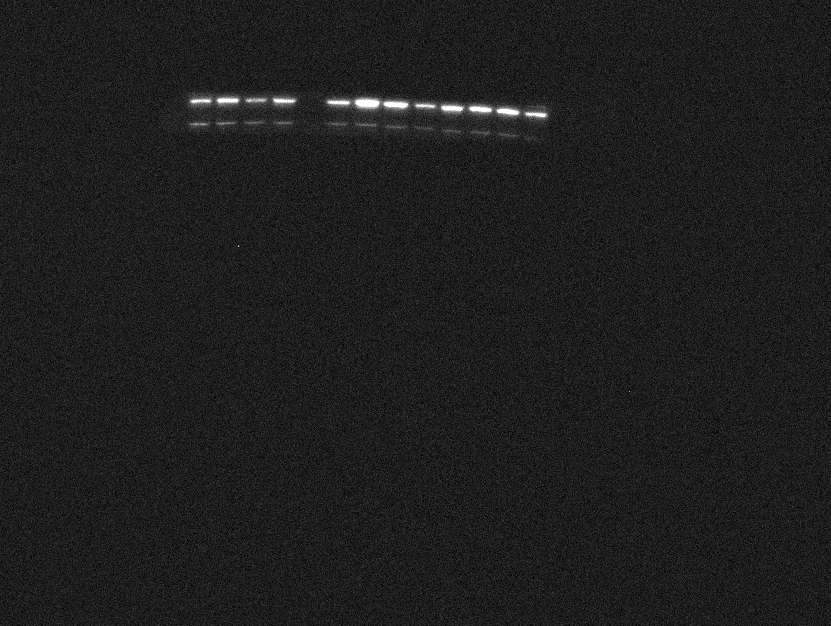

Supplement: Supplementary file 1 [file DataSheet_1.zip › WB1/2019-04-06_Wnt cp/2019-04-06_Wnt cp 16bit.png]

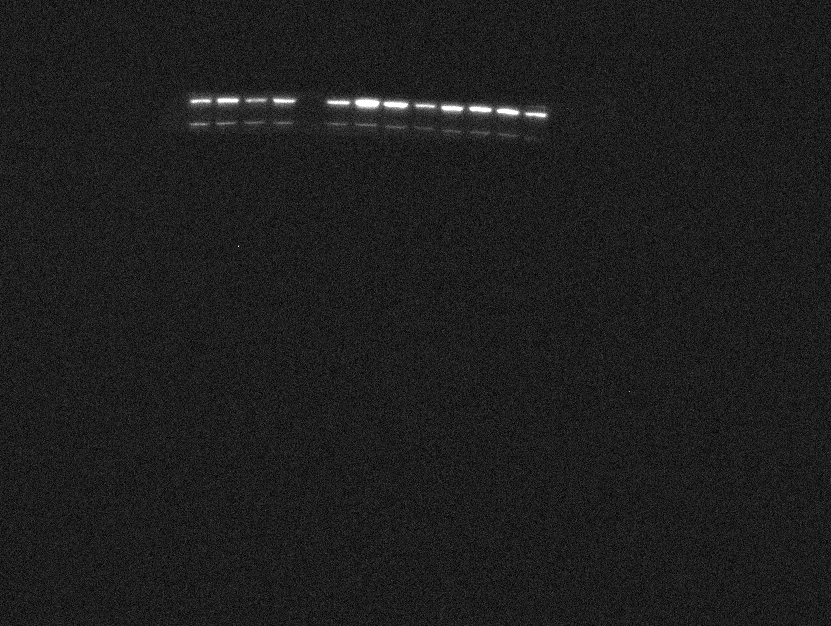

Supplement: Supplementary file 1 [file DataSheet_1.zip › WB1/2019-04-06_Wnt cp/2019-04-06_Wnt cp.tif]

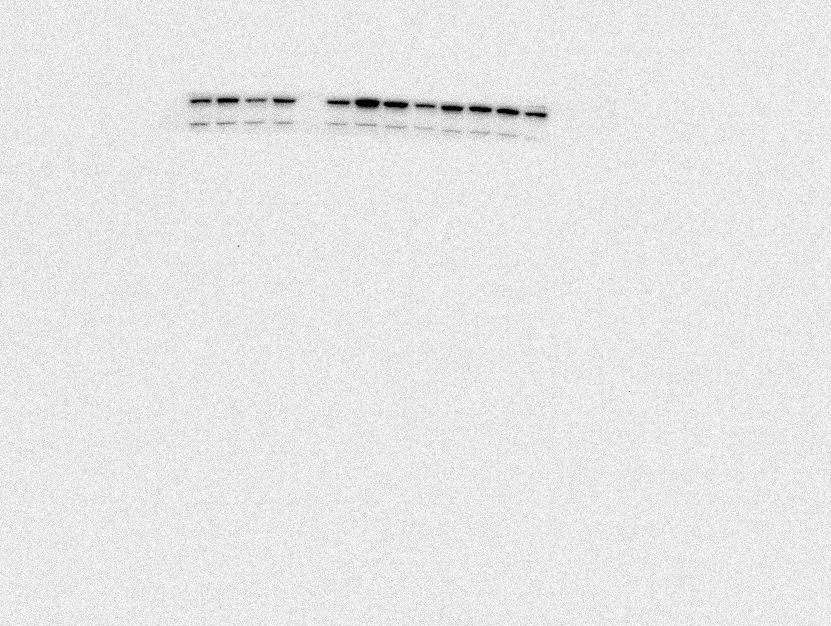

Supplement: Supplementary file 1 [file DataSheet_1.zip › WB1/2019-04-06_Wnt cp/2019-04-06_Wnt cp_8bit.png]

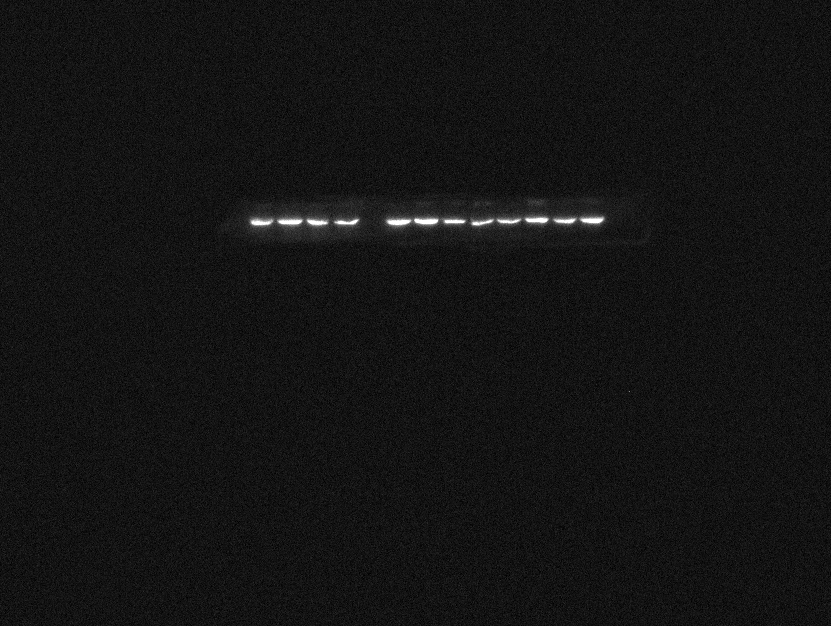

Supplement: Supplementary file 1 [file DataSheet_1.zip › WB1/2019-04-06_Wnt cp/2019-04-06_b act cp/2019-04-06_b act cp_1_16bit.png]

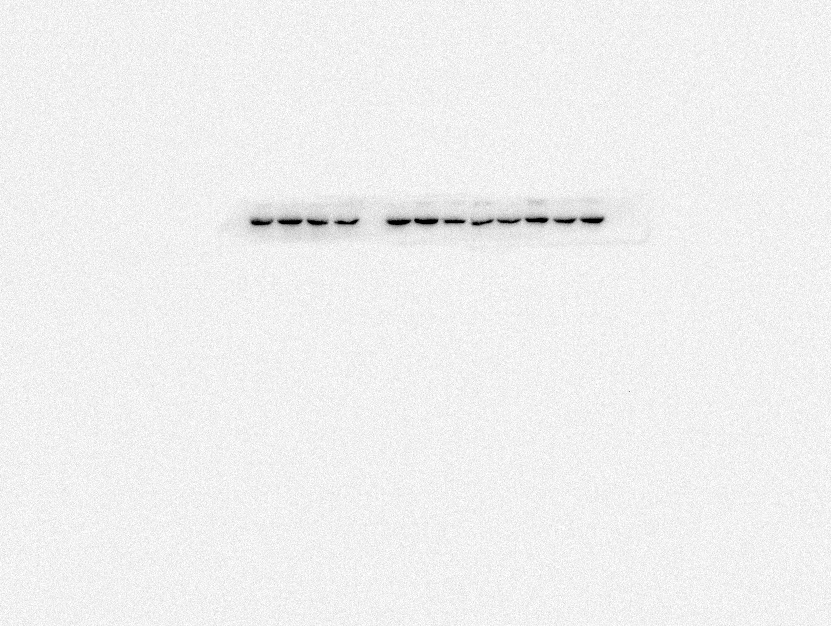

Supplement: Supplementary file 1 [file DataSheet_1.zip › WB1/2019-04-06_Wnt cp/2019-04-06_b act cp/2019-04-06_b act cp_8bit.png]

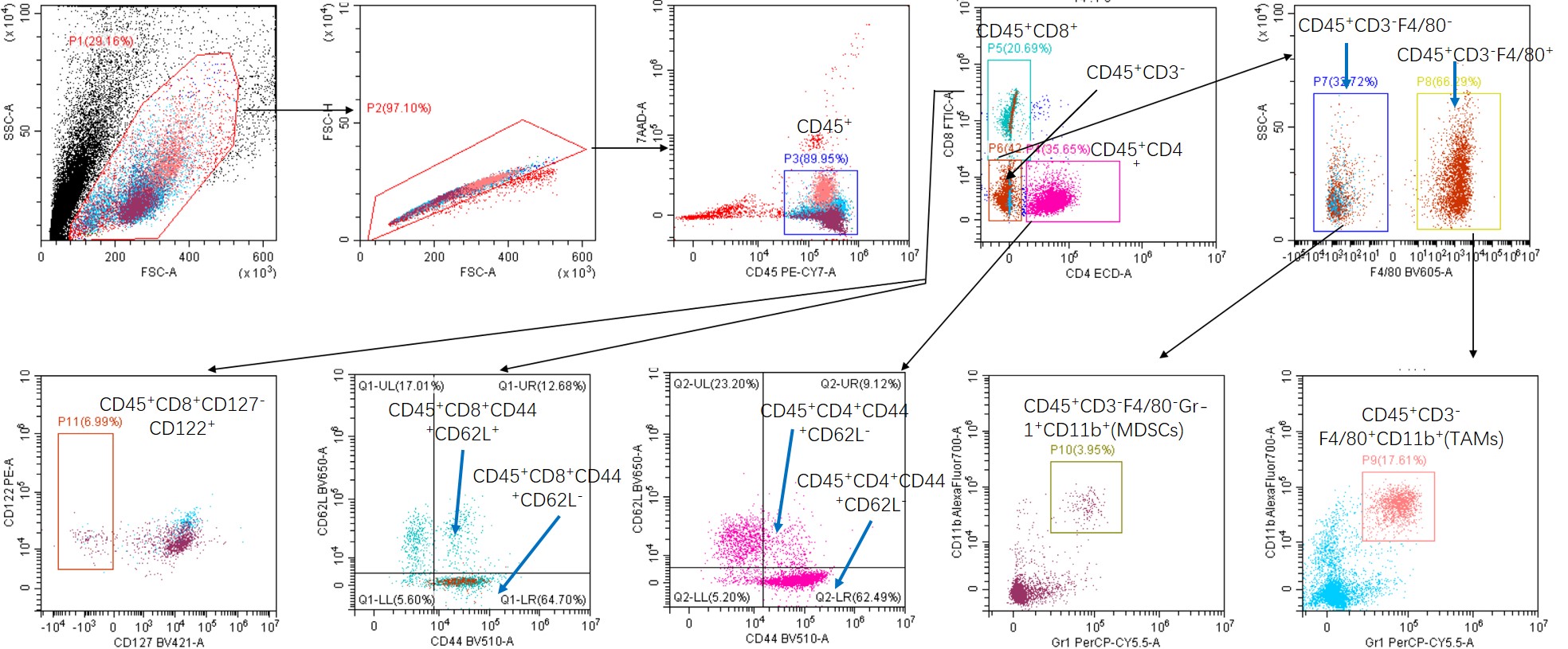

Supplement: Supplementary file 2 [file Image_1.jpeg]

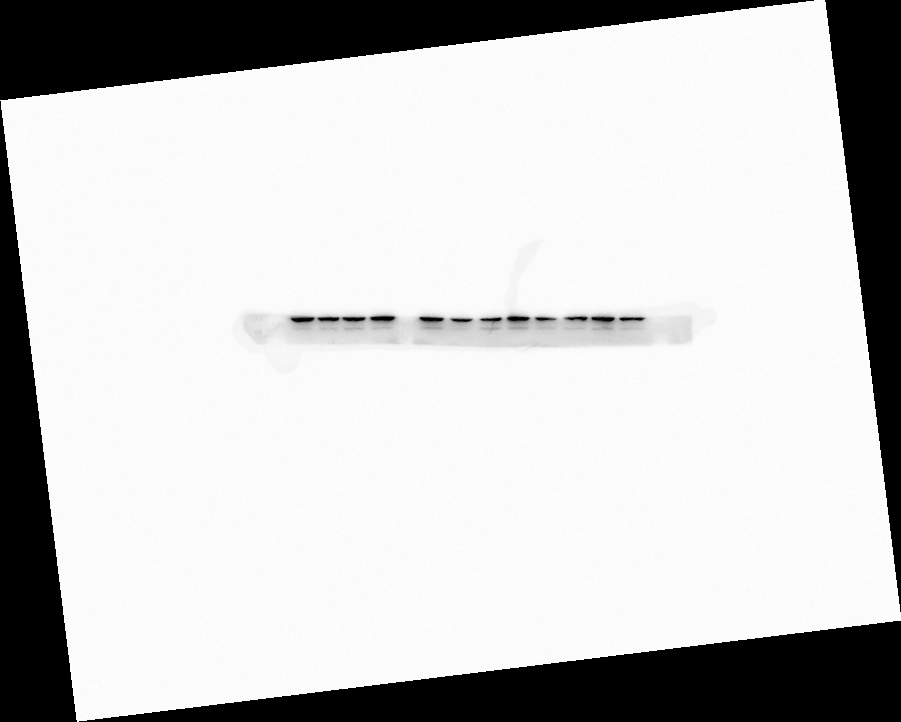

Supplement: Supplementary file 3 [file DataSheet_2.zip › WB2/2019-04-09_vegf cp/2019-04-09_b-act/2019-04-09_b-act- 8 bits 1.tif]

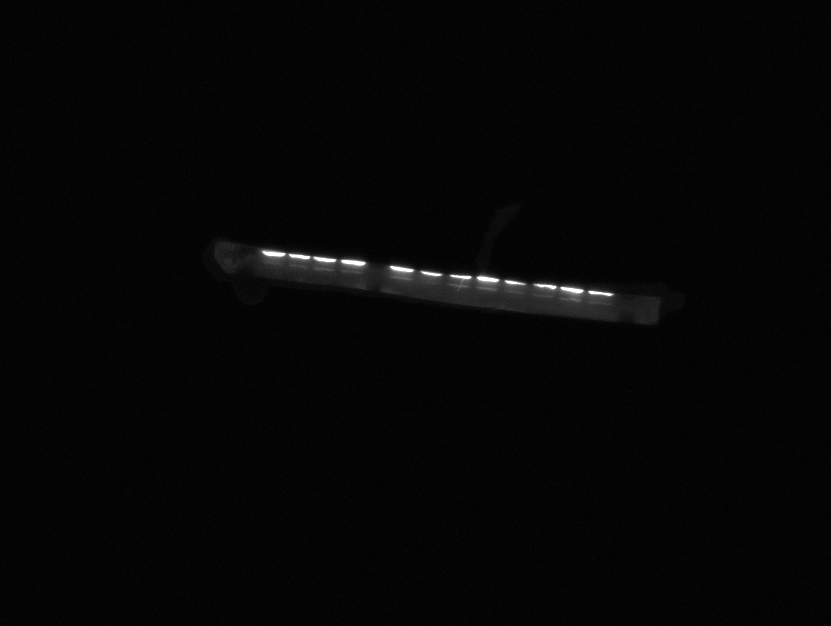

Supplement: Supplementary file 3 [file DataSheet_2.zip › WB2/2019-04-09_vegf cp/2019-04-09_b-act/2019-04-09_b-act_16bit.png]

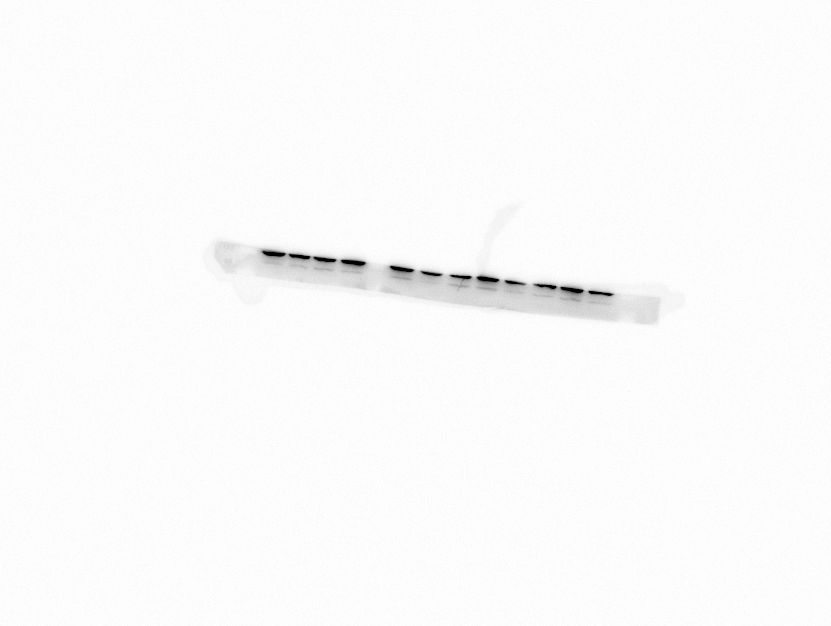

Supplement: Supplementary file 3 [file DataSheet_2.zip › WB2/2019-04-09_vegf cp/2019-04-09_b-act/2019-04-09_b-act_8bit.png]

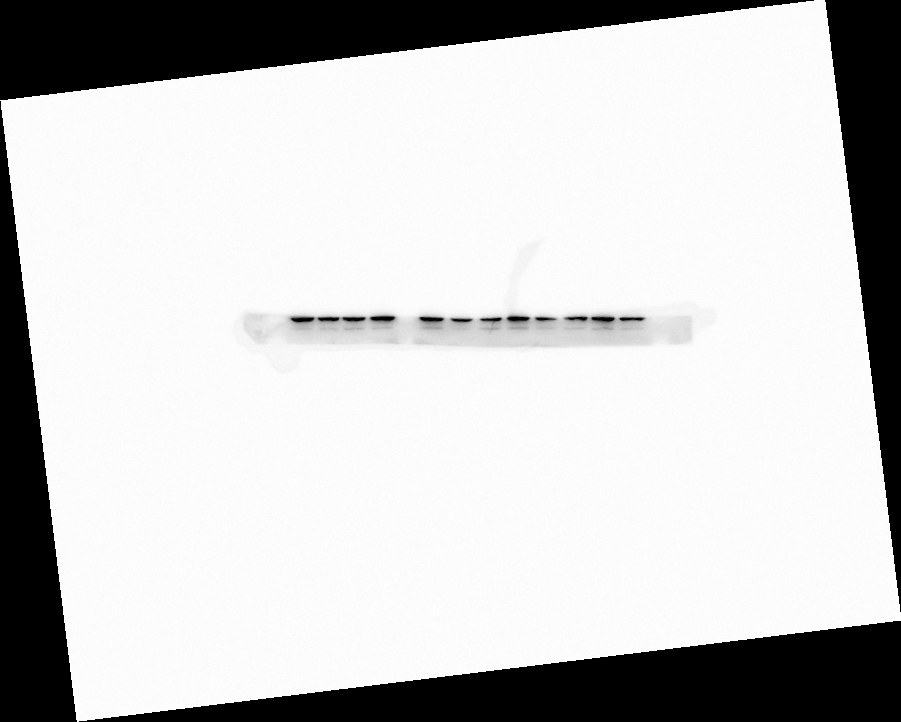

Supplement: Supplementary file 3 [file DataSheet_2.zip › WB2/2019-04-09_vegf cp/2019-04-09_b-act/2019-04-09_b-act_8bit.tif]

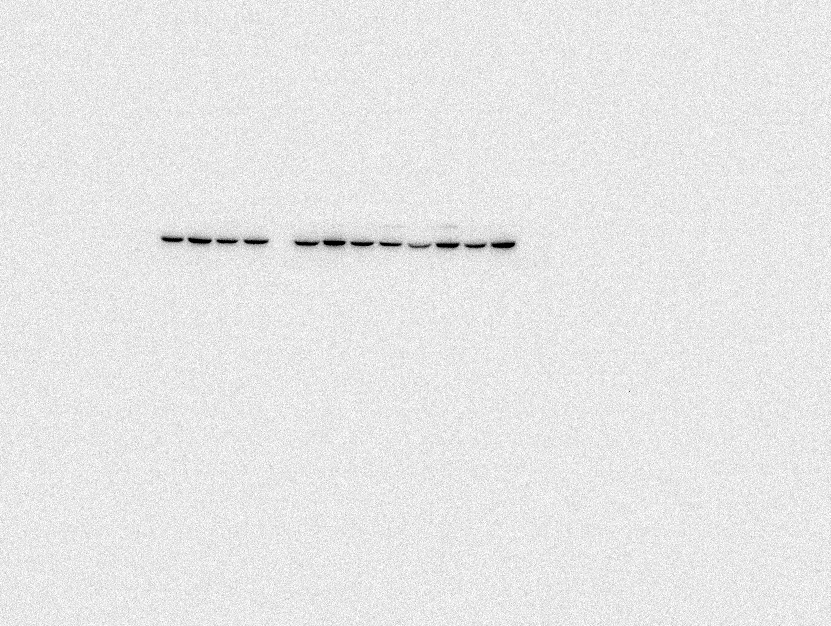

Supplement: Supplementary file 3 [file DataSheet_2.zip › WB2/2019-04-09_vegf cp/2019-04-09_vegf cp_8bit.png]

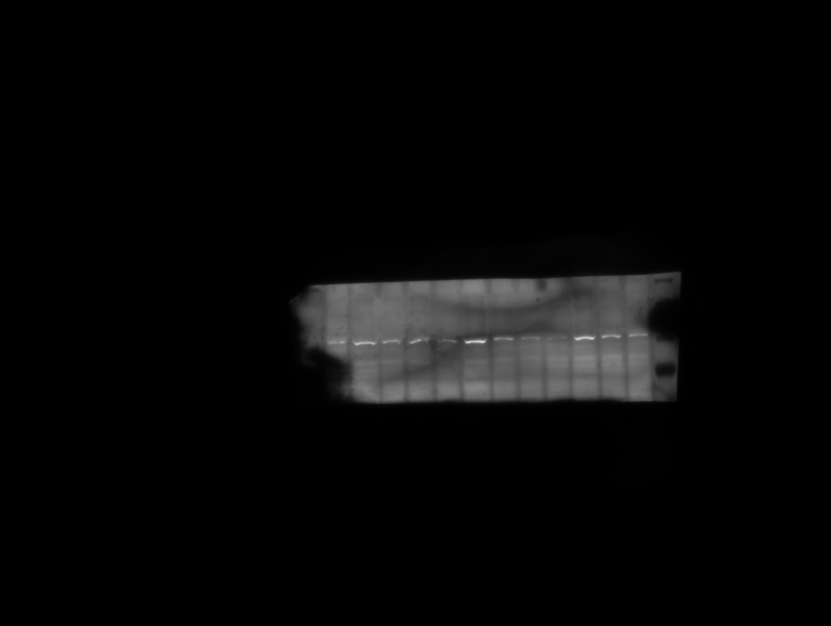

Supplement: Supplementary file 3 [file DataSheet_2.zip › WB2/2019-11-09_Frz/2019-11-09_Frz_1_16bit.png]

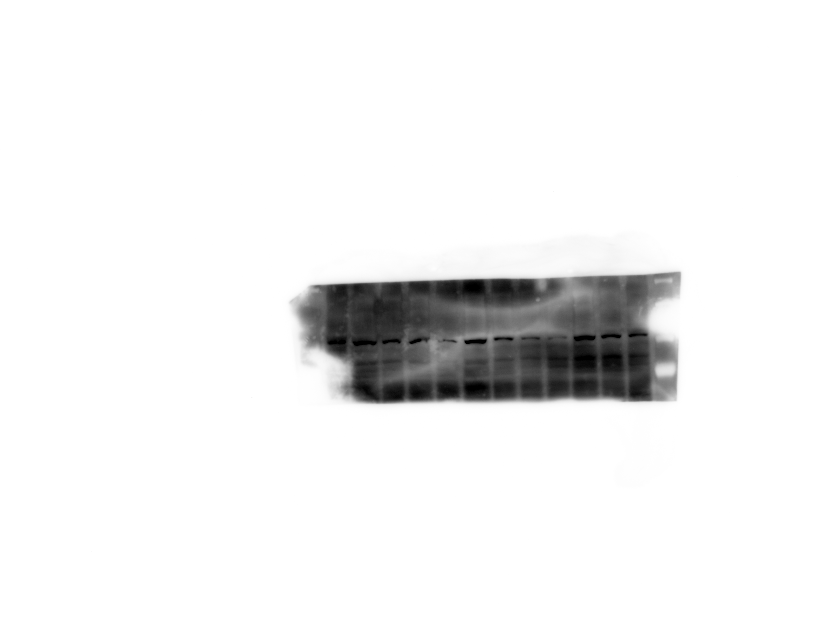

Supplement: Supplementary file 3 [file DataSheet_2.zip › WB2/2019-11-09_Frz/2019-11-09_Frz_8bit.png]

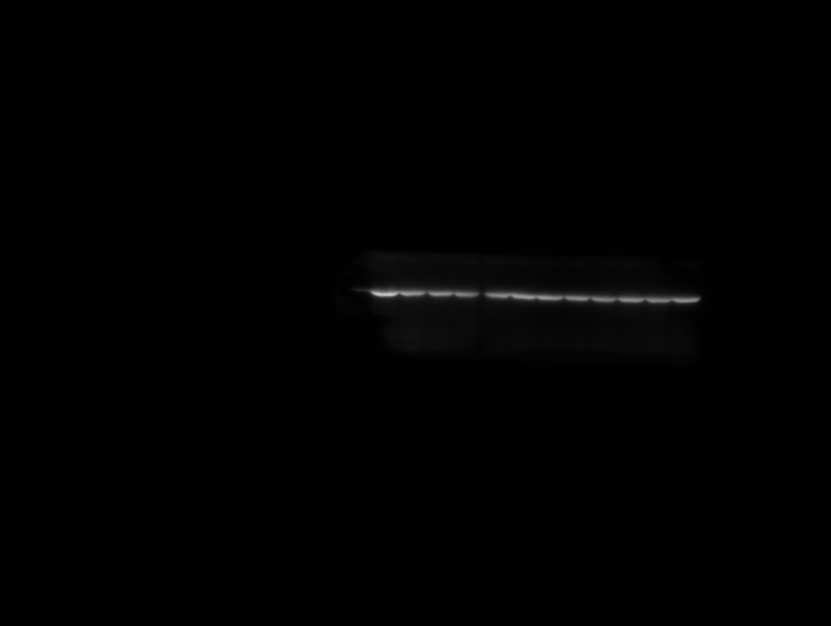

Supplement: Supplementary file 3 [file DataSheet_2.zip › WB2/2019-11-09_Frz/2019-11-10cp-bactin/2019-11-10cp-bactin_1_16bit.png]

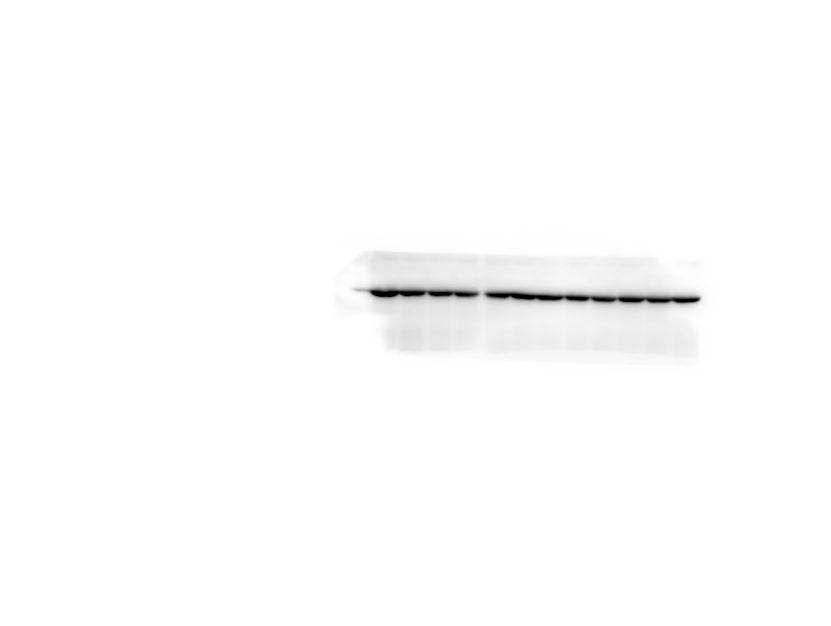

Supplement: Supplementary file 3 [file DataSheet_2.zip › WB2/2019-11-09_Frz/2019-11-10cp-bactin/2019-11-10cp-bactin_8bit.png]

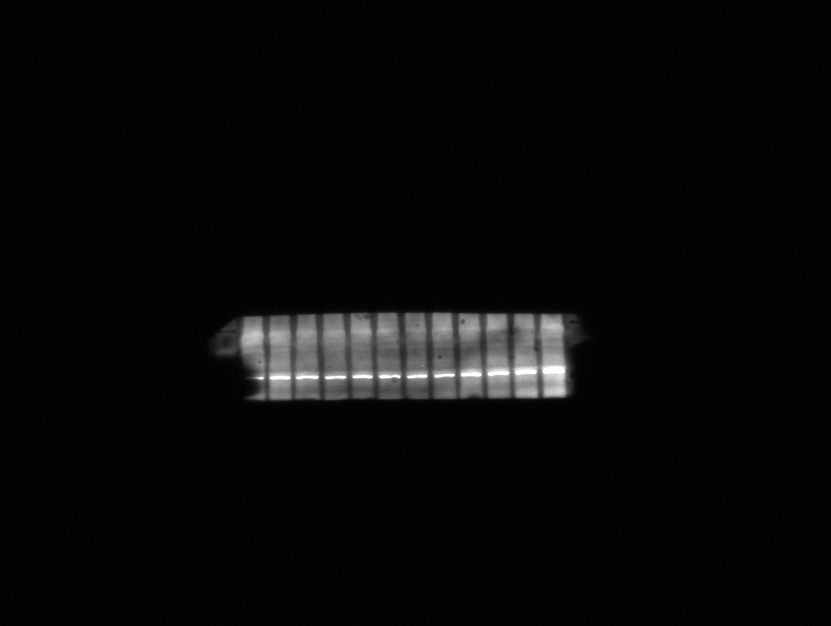

Supplement: Supplementary file 3 [file DataSheet_2.zip › WB2/2019-11-09_Pakt/2019-11-09_Akt cp/2019-11-09_Akt cp_1_16bit.png]

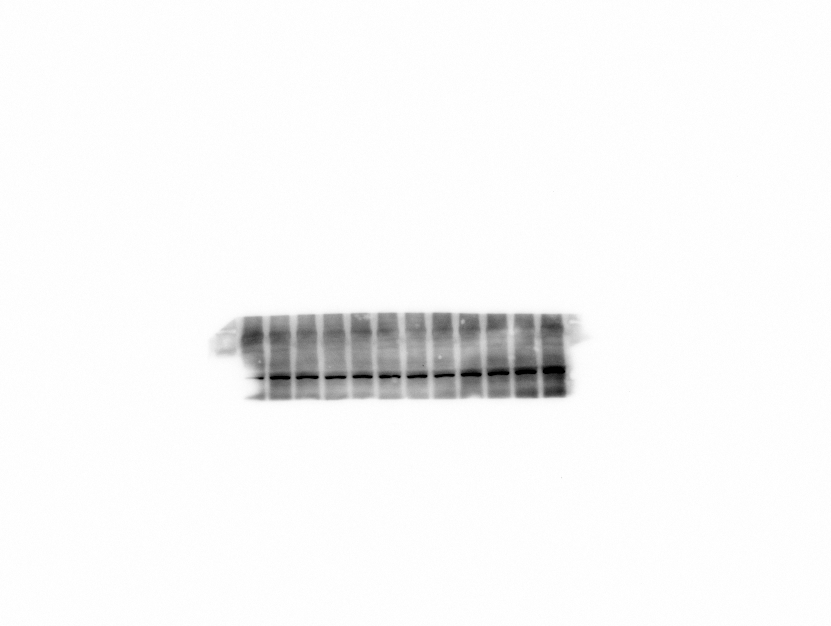

Supplement: Supplementary file 3 [file DataSheet_2.zip › WB2/2019-11-09_Pakt/2019-11-09_Akt cp/2019-11-09_Akt cp_8bit.png]

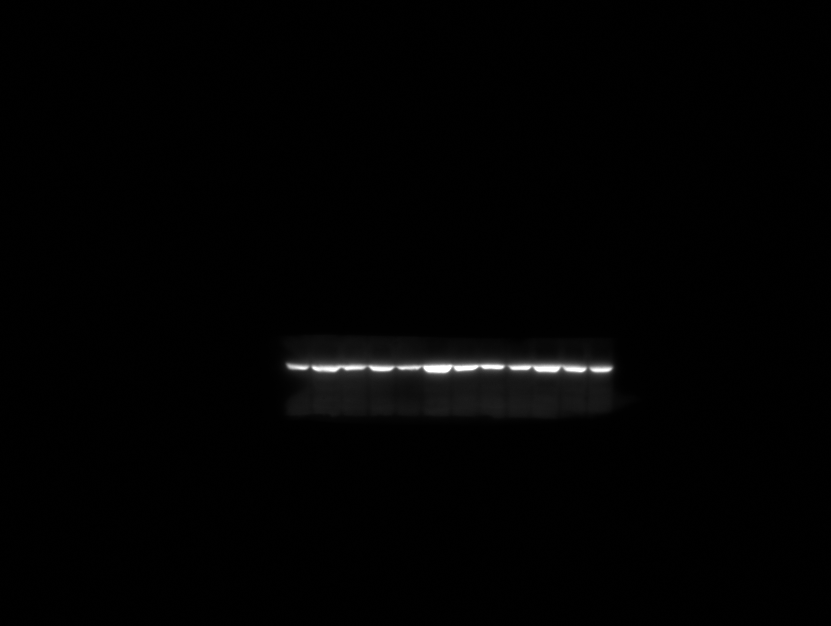

Supplement: Supplementary file 3 [file DataSheet_2.zip › WB2/2019-11-09_Pakt/2019-11-09_Pakt_1_16bit.png]

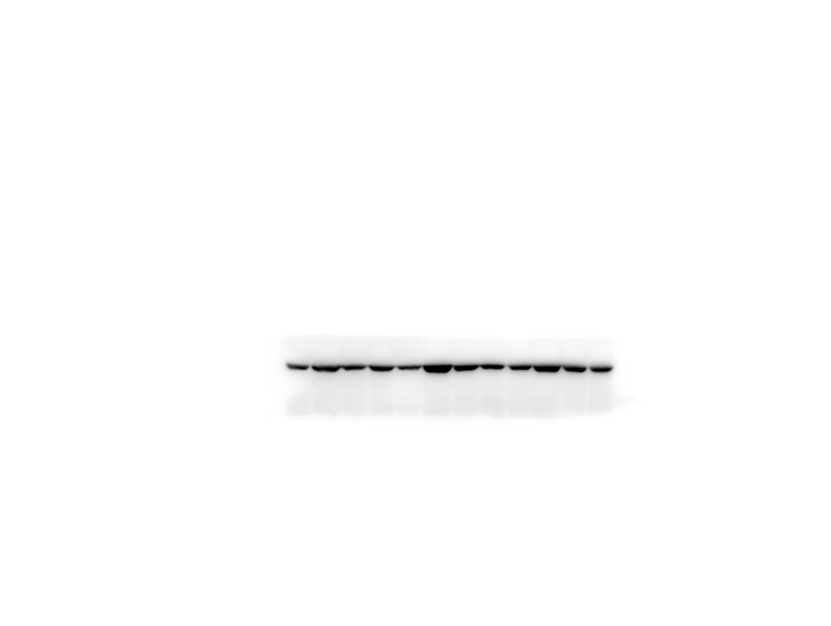

Supplement: Supplementary file 3 [file DataSheet_2.zip › WB2/2019-11-09_Pakt/2019-11-09_Pakt_8bit.png]

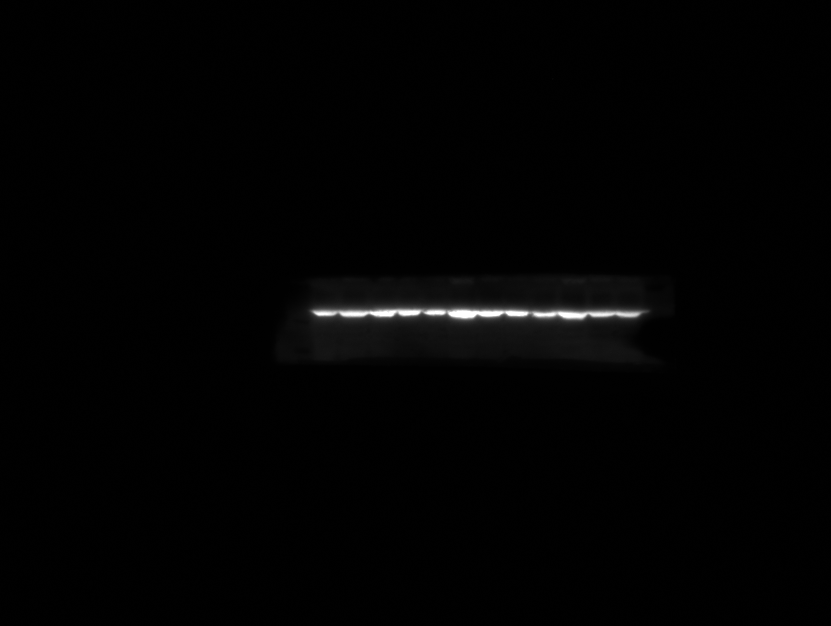

Supplement: Supplementary file 3 [file DataSheet_2.zip › WB2/2019-11-09_Psrc/2019-11-09_Psrc_1_16bit.png]

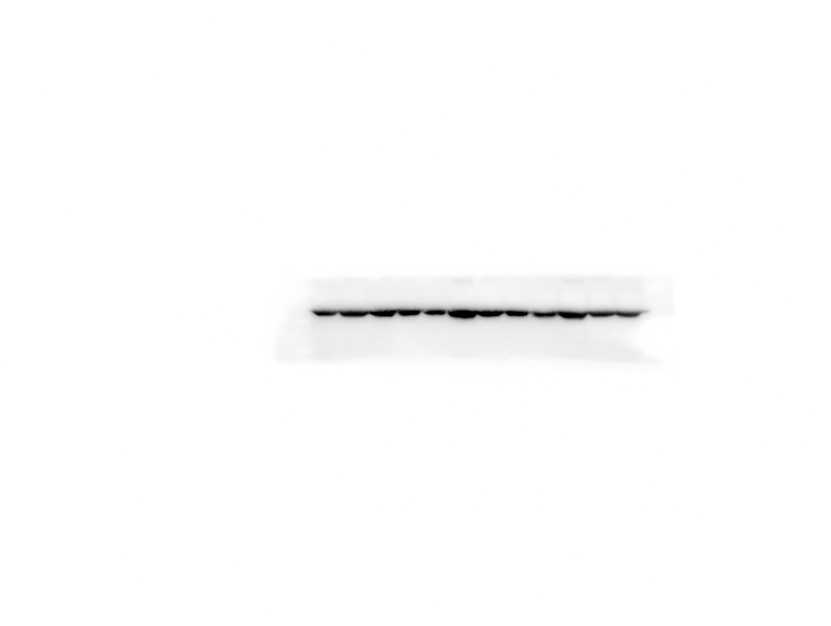

Supplement: Supplementary file 3 [file DataSheet_2.zip › WB2/2019-11-09_Psrc/2019-11-09_Psrc_8bit.png]

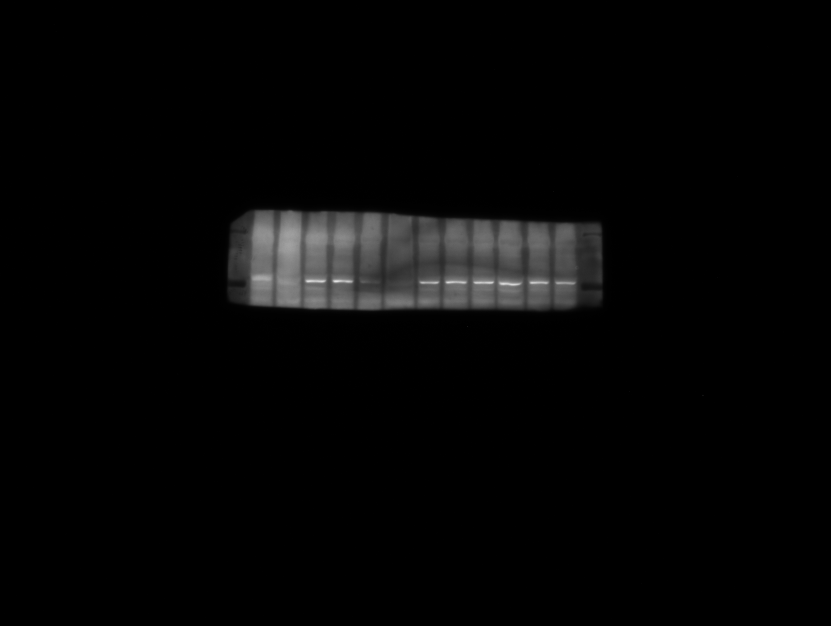

Supplement: Supplementary file 3 [file DataSheet_2.zip › WB2/2019-11-09_Psrc/2019-11-09_Src cp/2019-11-09_Src cp_1_16bit.png]

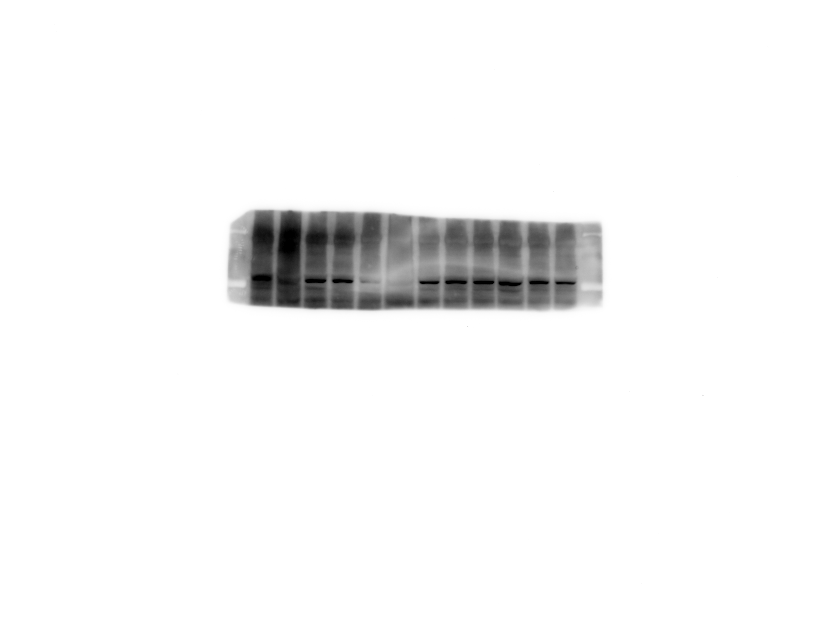

Supplement: Supplementary file 3 [file DataSheet_2.zip › WB2/2019-11-09_Psrc/2019-11-09_Src cp/2019-11-09_Src cp_8bit.png]

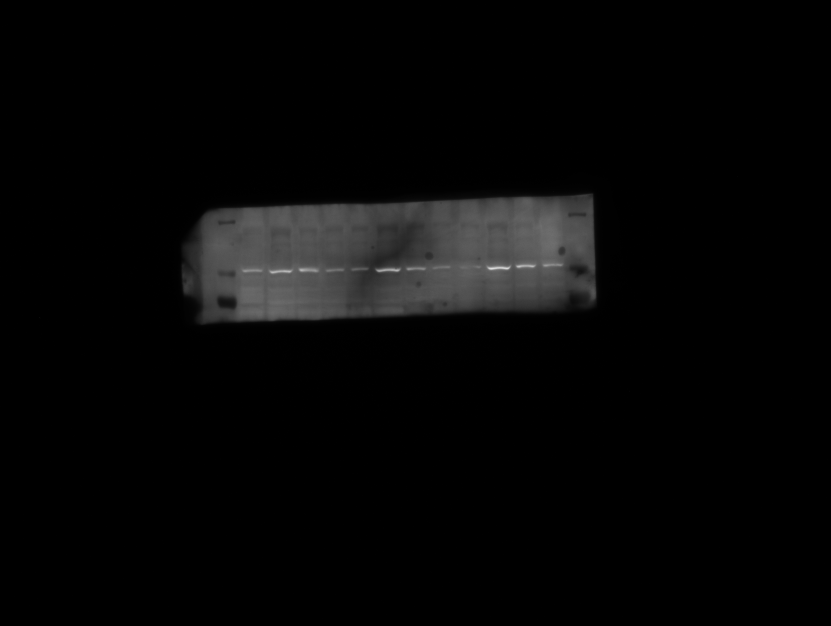

Supplement: Supplementary file 3 [file DataSheet_2.zip › WB2/2019-11-09_bcaten/2019-11-09_bcaten_1_16bit.png]

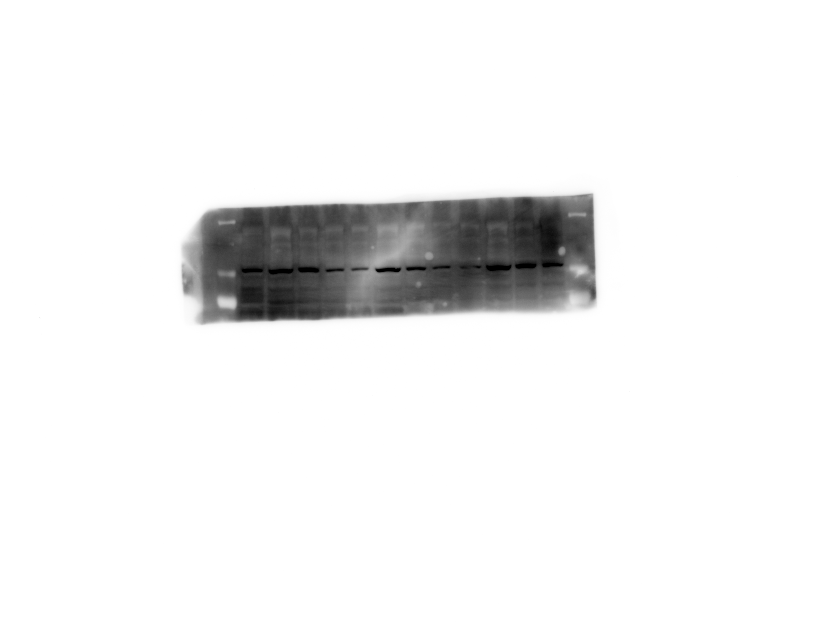

Supplement: Supplementary file 3 [file DataSheet_2.zip › WB2/2019-11-09_bcaten/2019-11-09_bcaten_8bit.png]

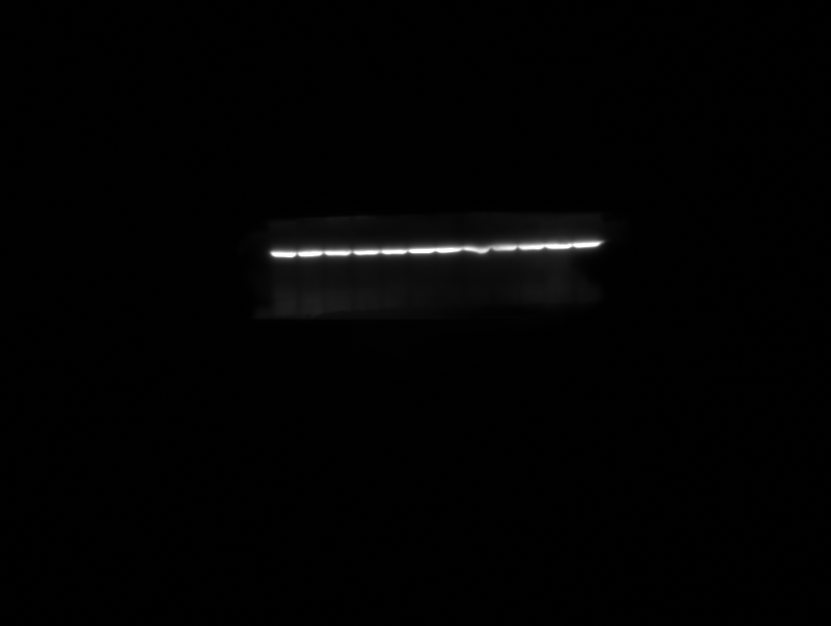

Supplement: Supplementary file 3 [file DataSheet_2.zip › WB2/2019-11-09_bcaten/2019-11-10_bactin-cp/2019-11-10_bactin-cp_1_16bit.png]

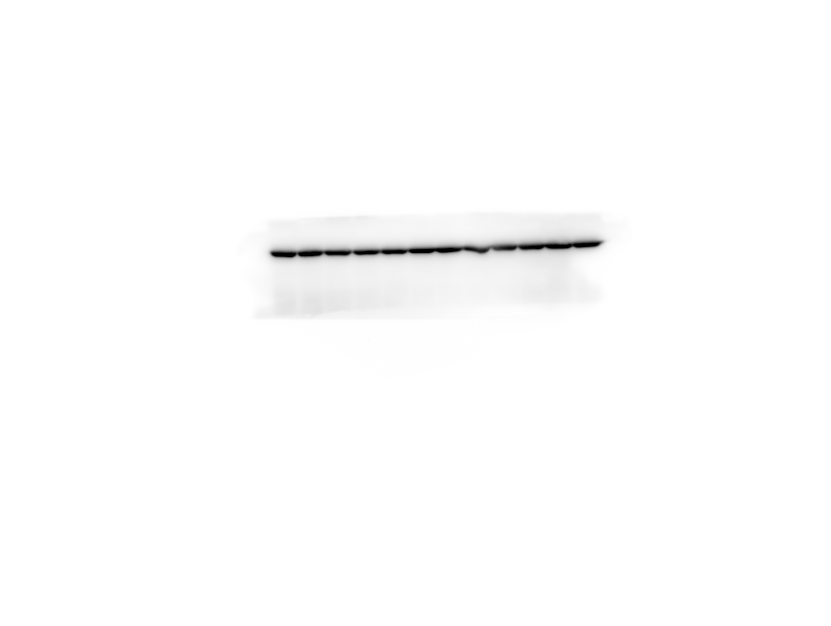

Supplement: Supplementary file 3 [file DataSheet_2.zip › WB2/2019-11-09_bcaten/2019-11-10_bactin-cp/2019-11-10_bactin-cp_8bit.png]
